# Tuning LiBH_4_ for Hydrogen Storage: Destabilization, Additive, and Nanoconfinement Approaches

**DOI:** 10.3390/molecules25010163

**Published:** 2019-12-31

**Authors:** Julián Puszkiel, Aurelien Gasnier, Guillermina Amica, Fabiana Gennari

**Affiliations:** Consejo Nacional de Investigaciones Científicas y Técnicas, CONICET—Instituto Balseiro (UNCuyo and CNEA), Departamento Fisicoquímica de Materiales, Gerencia de Investigación Aplicada, Centro Atómico Bariloche (CNEA), R8402AGP S. C. de Bariloche, Río Negro, Argentina; aurelien.gasnier@gmail.com (A.G.); guillerminaamica@gmail.com (G.A.); gennari@cab.cnea.gov.ar (F.G.)

**Keywords:** borohydrides, hydrogen, storage, destabilization, additive, rare earth, nanoconfinement

## Abstract

Hydrogen technology has become essential to fulfill our mobile and stationary energy needs in a global low–carbon energy system. The non-renewability of fossil fuels and the increasing environmental problems caused by our fossil fuel–running economy have led to our efforts towards the application of hydrogen as an energy vector. However, the development of volumetric and gravimetric efficient hydrogen storage media is still to be addressed. LiBH_4_ is one of the most interesting media to store hydrogen as a compound due to its large gravimetric (18.5 wt.%) and volumetric (121 kgH_2_/m^3^) hydrogen densities. In this review, we focus on some of the main explored approaches to tune the thermodynamics and kinetics of LiBH_4_: (I) LiBH_4_ + MgH_2_ destabilized system, (II) metal and metal hydride added LiBH_4_, (III) destabilization of LiBH_4_ by rare-earth metal hydrides, and (IV) the nanoconfinement of LiBH_4_ and destabilized LiBH_4_ hydride systems. Thorough discussions about the reaction pathways, destabilizing and catalytic effects of metals and metal hydrides, novel synthesis processes of rare earth destabilizing agents, and all the essential aspects of nanoconfinement are led.

## 1. Introduction

**Figure 1:**
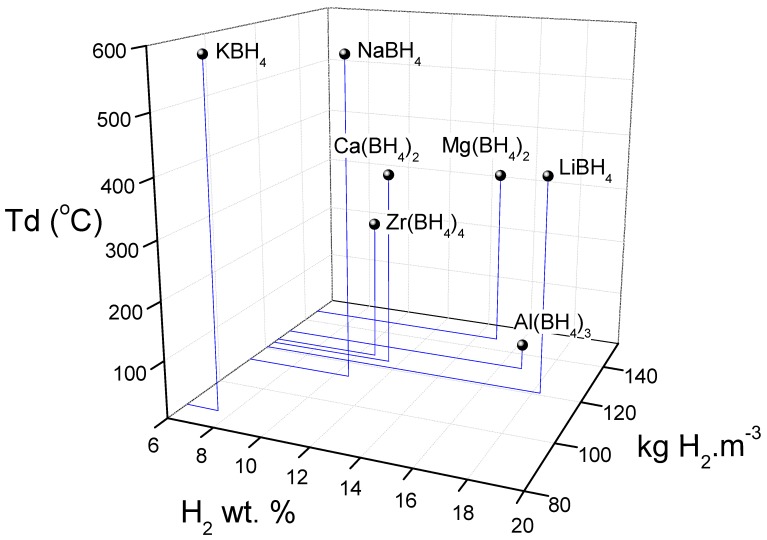
Hydrogen gravimetric capacity (wt.%), volumetric capacity (kg H_2_·m^−3^), and decomposition temperature (Td) of alkali, alkali earth, Zr, and Al borohydrides.

LiBH_4_ has been exhaustively investigated as a hydrogen storage medium owing to its large gravimetric (18.5 wt.%) and volumetric (121 kgH_2_/m^3^) hydrogen densities. However, the hydrogen contained in the LiBH_4_ is not entirely available since its decomposition leads to the formation of LiH, free boron, and just 13.8 wt.% of hydrogen is released in the range of 380 °C to 680 °C under 1 bar of pressure. Figure 1 shows the volumetric and gravimetric hydrogen contained and decomposition temperature (Td) of the most relevant alkali and alkali earth borohydrides, as well as Zr and Al borohydrides, which have been a matter of exhaustive research. Rb, Cs, Fr, Sr, Ba, and Ra borohydrides are not included owing to either low capacity (<6 wt.%) or Td higher than 600 °C. In the case of Be borohydride, with ~21 wt.% H_2_, it is not included because Be is extremely toxic. As seen, LiBH_4_ contains the highest gravimetric capacity but has a relatively high decomposition temperature [1,2,3,4,5,6,7]. However, most of the hydrogen release from LiBH_4_ occurs above 500 °C and at a slow rate. The rehydrogenation process from LiH and free B requires harsh temperature and hydrogen pressure conditions over 600 °C and 100 bar [1]. These hydrogen absorption and desorption characteristics preclude the employment of LiBH_4_ as a hydrogen storage material, mainly for mobile applications.

Several reviews including borohydrides as potential hydrogen storage materials have been published [2,3,4,5,6,7,8,9,10,11,12,13,14,15,16,17,18,19,20,21,22,23,24,25,26,27]. They focus on different features such as synthesis [3,7,8,12,15,19,21,22,23,24,25], crystal structure [3,8,9,11,15,18,19,21,22,26], kinetic and thermodynamic features [2,3,4,10,11,16,17,20,21,22,23,24,25,26], tailoring their thermodynamic and kinetic behavior [3,4,5,6,7,10,15,16,17,20,21,23,24,25,26], and nanoconfinement [4,10,13,14,15,17,20,21,24,25,26,27]. Recently, a review devoted to LiBH_4_ has been released, describing five different approaches to tune LiBH_4_: nanoengineering, catalyst modification, ion substitution, destabilization, and high-energy milling [27].

Despite the vast information about borohydrides, a fresh and different outlook about a promising borohydride such as LiBH_4_ provides a clear input for the understanding and future improvements towards its practical application. Therefore, this review is about some of the main explored approaches to tuning LiBH_4_, taking into account the novel contributions done by the authors of this work (Departamento Fisicoquímica de Materiales, CAB–CNEA–CONICET, Río Negro, Argentina). At the beginning, in the second section, we describe the concept of thermodynamic destabilization for one of the most attractive so-called reactive hydride composites, 2LiBH_4_:MgH_2_, covering the evolution of the investigations into the hydrogenation and dehydrogenation reaction pathways, and the enhancement of the kinetic behavior through the addition of transition metals and transition metal compounds. Then, the third section is about the thermodynamic destabilization behavior, and kinetic enhancements of the hydride systems composed of metal and binary hydride added LiBH_4_. This section describes the theoretical predictions as well as experimental results obtained from the effects of an extensive list of metals (Al, Mg, Ti, V, Cr, Sc, Ni, Ca, In and Fe) and binary hydrides (AlH_3_, TiH_2_, VH_2_, ScH_2_, CrH_2_, CaH_2,_ and MgH_2_) on LiBH_4_. Then, the fourth section is devoted to the combination of LiBH_4_ with rare earth (RE) hydrides. In this regard, different synthesis routes of non-commercial RE hydrides are presented, and the destabilization and kinetic effects of the addition of RE hydrides to LiBH_4_ are discussed. Finally, in the fifth section, we focus on the utilization of nanoconfinement to tailor the thermodynamic stability and kinetic behavior of LiBH_4_. A detailed discussion about the experimental techniques to characterize the nanoconfined hydride systems, different nanoconfinement approaches, types of matrix, and performances of the nanoconfined LiBH_4_ based hydride systems are exposed.

## 2. Destabilized MgH_2_-2LiBH_4_ System: Li-RHC

**Figure 2:**
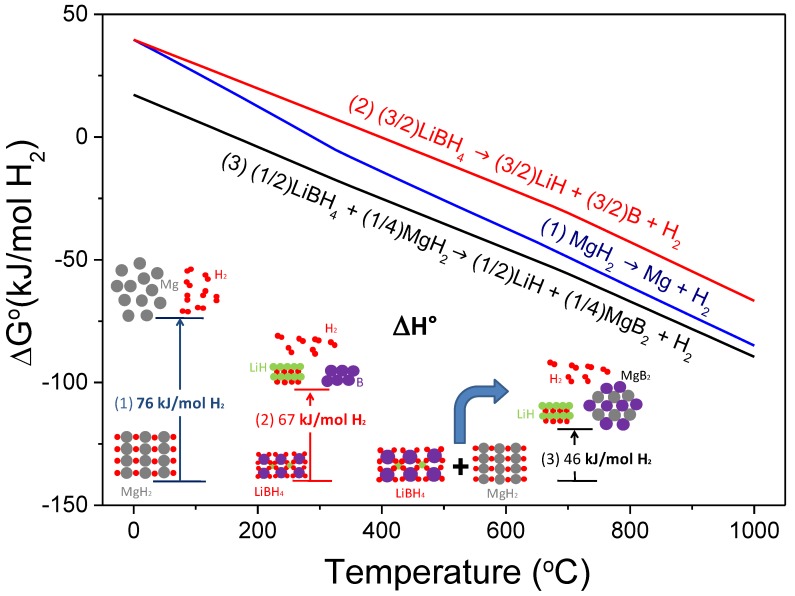
Free energy per mol of H_2_ as a function of temperature and the standard enthalpy of a reaction of the hydride system 2LiBH_4_ + MgH_2_.

One of the most attractive destabilized hydride systems is the stoichiometric mixture MgH_2_:2LiBH_4_, or the so-called Li-RHC system (RHC: Reactive Hydride Composite). The destabilization concept refers to Reaction (1), through which the theoretical overall reaction enthalpy in standard conditions is notably reduced to 46 kJ mol^−1^ H_2_ in comparison with MgH_2_ (76 kJ mol^−1^ H_2_) and LiBH_4_ (67 kJ mol^−1^ H_2_; for decomposition to LiH, B, and H_2_) [28]. Destabilizing both hydrides by the RHC concept means the reduction of the reaction enthalpy by the exothermal formation of MgB_2_ upon dehydrogenation. Such a low enthalpy value leads to a dehydrogenation temperature of about 40 °C under 1 bar of H_2_, considering the entropy value of ~130 J mol^−1^ H_2_ K^−1^ for the change of hydrogen from the gas phase to the solid phase in a conventional metal-hydrogen system. Furthermore, Li-RHC has a theoretical hydrogen capacity of 11.4 wt.%. Figure 2 depicts the free energy per mol of H_2_ and the standard enthalpy of reaction per mol of H_2_ resulting from the mutual destabilization of LiBH_4_ and MgH_2_, calculated with HSC software [28].

Vajo et al. [29] in 2005 first published a work about the destabilized Li-RHC system, and Barkohrdarian et al. [30] patented the concept of RHC in 2006. In the first published work about Li-RHC, the hydride mixture was doped with 2–3 mol% TiCl_3_ to improve the kinetic behavior, and pressure composition isotherm measurements (PCIs) were done in the range of 315 °C to 400 °C. The hydrogenation experimental enthalpy amounted to 40.5 kJ mol^−1^ H_2_, while the entropy value was 81.3 J mol^−1^ H_2_ K^−1^ [29]. These values provide a dehydrogenation temperature of 225 °C at 1 bar. Also noteworthy, the entropy value for the Li-RHC system is different from the one for the metal-hydrogen systems. This phenomenon is related to the [BH_4_]^−^ cluster configuration upon hydrogen interaction [31]. Even at the beginning of the boom of Li-RHC, essential hydrogen storage properties obtained from thermodynamic measurements such as decomposition temperature (T_d_: 225 °C) and hydrogen capacity (8–10 wt.%) exposed a diminished potential in comparison with the predicted ones (T_d_ ~ 40 °C and 11.4 wt.%).

PCIs are usually measured in Sieverts kind devices and consist of providing/taking small amounts of hydrogen to/from a hydride forming material/hydride kept at a constant temperature, letting the reaction reach an equilibrium, and recording the change of equilibrium pressure as a function of the absorbed/desorbed hydrogen capacity [31,32].

MgH_2(s)_ + 2LiBH_4(s)_ ⇆ MgB_2(s)_ + 2LiH_(s)_ + 4H_2(g)_ (1)

Investigations into reaction pathways under different temperatures, hydrogen pressures, and stoichiometric compositions were carried out in order to understand the behavior of the Li-RHC and try to optimize its operative conditions. These investigations were focused mainly on the dehydrogenation pathways [33,34,35,36,37,38,39,40]. In 2007, Bösenberg et al. [33] first reported the global hydrogenation and dehydrogenation reaction mechanisms of Li-RHC under dynamic conditions. Upon hydrogenation from MgB_2_ + 2LiH, both LiBH_4_ and MgH_2_ are simultaneously formed at the early step of the process following one reaction step at relatively mild conditions, i.e., 250–300 °C and 50 bar of H_2_. Thus, this indicates that there is a mutual destabilization effect of both hydrides during hydrogenation. Upon dehydrogenation, however, a two-step reaction was observed: first, the dehydrogenation of MgH_2_ and then the decomposition of LiBH_4_ with the formation of MgB_2_ and LiH, and the release of H_2_. The two-step reaction mechanism accounted for kinetic constraints, leading to high dehydrogenation temperatures over 400 °C and under 3–5 bar of H_2_. Pinkerton et al. [34] studied the thermodynamic and kinetic stable conditions for the reversible hydrogen storage of TiCl_3_-catalyzed Li-RHC. They established an H_2_ pressure–temperature phase diagram for the dehydrogenation process from vacuum to 5 bar and from 250 °C to 500 °C. At low pressure (<3 bar) and high temperature (>400 °C), MgH_2_ decomposes to Mg and H_2_, and the direct decomposition of LiBH_4_ is thermodynamically and kinetically favored towards LiH and amorphous B. Under pressures between 3 and 5 bar and temperatures from 280 °C to 450 °C, MgH_2_ and LiBH_4_ decompose independently, but the formation of MgB_2_ and LiH are promoted as solid final products. These results were in agreement with the work of Bösenberg et al. [33]. It was found that the formation of amorphous boron under low hydrogen pressure upon dehydrogenation precludes the rehydrogenation of Li-RHC at milder temperature and pressure conditions than the required for pristine LiBH_4_ (~600–700 °C and ~100–200 bar) [1,41]. Barkhordarian et al. [42] proposed that the formation of MgB_2_ instead of free B markedly reduces the activation barrier for the formation of the [BH_4_]^−^ clusters. Investigations into the effects of the hydrogen backpressure on the dehydrogenation of Li-RHC were also reported by Nakagawa et al. [43]. They also found that under an inert gas atmosphere, the free B and metallic Mg are formed upon dehydrogenation.

On the contrary, conditions such as 5 bar of H_2_ backpressure and 400 °C promote the formation of MgB_2_ from the solid–liquid interaction between solid Mg and liquid LiBH_4_; between 250 °C and 280 °C, LiBH_4_ melts [44]. An attempt to achieve reversibility in the solid-state was made with a 2LiH + MgB_2_ mixture milled for 120 h under high-energy conditions [35]. The nanostructured particles, higher lattice microstrain, and large surface areas of the reactants allowed faster hydrogenation and dehydrogenation rates below the melting temperature of LiBH_4_ at 265 °C, achieving 8.3 wt.% of hydrogen capacity. Upon hydrogenation under non-isothermal conditions and 90 bar, the reaction path went via the mutual formation of MgH_2_ and LiBH_4_. However, upon dehydrogenation under non-isothermal conditions and vacuum, the two-step reaction with the formation of MgB_2_ was reported. This behavior might have occurred because of the 5 h of isothermal conditions at 265 °C after the temperature ramp.

Differential scanning calorimetry (DSC) was mainly utilized to study the effect of the hydrogen backpressure on the Li-RHC system. DSC is a thermoanalytical technique in which the difference of heat required to increase the temperature of a sample, as well as a reference, is recorded as a function of temperature. Thus, the heat released or required for exothermal and endothermal processes, respectively, as well as the range of temperature at which the thermal processes occur, and the temperature at the maximum rate of the processes, among the most important features, can be measured [45].

Yu et al. proposed other dehydrogenation mechanisms working with 1LiBH_4_:4MgH_2_ under vacuum and non-isothermal conditions up to 600 °C [36]. A three-step reaction path was proposed: First, the dehydrogenation of MgH_2_ at about 360 °C. Second, the decomposition of LiBH_4_ into LiH, B, and H_2_ occurs at about 405 °C. Finally, the reaction among Mg, LiH, and B to form LiMg alloys and MgB_2_ at occurs at temperatures higher than 420 °C. This dehydrogenation mechanism was verified by in situ powder neutron diffraction with 1LiBD_4_:4MgD_2_ and under vacuum conditions. However, the in situ powder neutron diffraction with 2LiBD_4_:1MgD_2_ under 1 bar of initial pressure led to the two-step reaction with the formation of LiD, MgB_2_, and release of deuterium [46]. Therefore, the stoichiometric composition and hydrogen backpressure upon dehydrogenation play the central role in the reaction path and reversibility of the Li-RHC system.

A detailed investigation into the effects of the dehydrogenation temperature and pressure for 2LiBH_4_:1MgH_2_ on the reaction paths was published by Bösenberg et al. in 2010 [37]. Combining in situ X-ray diffraction (XRD) and Fourier transform infrared spectroscopy (FT-IR), a temperature–pressure map under dynamic conditions was established. At a temperature higher than 450 °C and hydrogen backpressure lower than 3 bar, the observed hydrogenation mechanism underwent individual MgH_2_ and LiBH_4_ decomposition, leading to metallic Mg and free B. Nonetheless, at 400 °C and hydrogen backpressure of 5 bar, the dehydrogenation mechanism led to the formation of MgB_2_ and LiH. Theoretical calculations already predicted as thermodynamically favorable the formation of Li_2_B_12_H_12_ as an intermediate compound during the dehydrogenation of 2LiBH_4_:MgB_2_ [47], but it was not verified in the work of Bösenberg et al. [37].

In situ X-ray diffraction techniques allow us to understand the dependence of the gas–solid reaction involving crystalline phases on pressure and temperature conditions. Specially designed cells are used in synchrotron facilities to assure short acquisition times and deeper penetration of XRD [48].

Studies about the possible formation of intermediate species at different hydrogen backpressures were also performed [38,49,50,51]. Non-isothermal dehydrogenation experiments with 2LiBH_4_:1MgH_2_ composition, under 1 bar of helium, and in the range between 30 °C and 600 °C showed five thermal events associated with the reaction pathway [38]. As the main features, the individual decomposition of MgH_2_ (367 °C) and LiBH_4_ (427 °C) happened in the first and second thermal events, respectively. In the case of LiBH_4_, it was found that the second thermal event corresponded to the formation of Li_2_B_12_H_12_, LiH, and a tiny amount of hydrogen release. The third thermal event at 447 °C belonged to the decomposition of Li_2_B_12_H_12_ into LiH, B, and hydrogen release. This behavior was in accordance with the prediction of the theoretical calculations [47] and the thermodynamic stabilities given by the enthalpy values, namely 56 kJ mol^−1^ H_2_ for the decomposition of LiBH_4_ into Li_2_B_12_H_12_ + LiH + H_2_ [38], and 74 kJ mol^−1^ H_2_ for the decomposition of LiBH_4_ into free B + LiH + H_2_ [52]. The formation of a MgLi alloy accounted for the fourth thermal event at 527 °C. Finally, the formation of MgB_2_ occurred from the melted MgLi alloy (melting temperature: 587 °C) and/or metallic Mg, and free B during the fifth thermal event. Thus, the formation of Li_2_B_12_H_12_ as an intermediate of the decomposition of LiBH_4_ was experimentally verified. Additionally, the presence of the MgLi alloy and the subsequent formation of MgB_2_ as a final product were also seen at high temperatures in agreement with work from Walker et al. [36,46]. As the hydrogen backpressure was increased from 5 to 10 bar, the dehydrogenation occurred through the formation of Li_2_B_12_H_12_ as an intermediate and MgB_2_ as the final product. A small amount of LiBH_4_ decomposed to Li_2_B_12_H_12_. A notable reduction of the amount of Li_2_B_12_H_12_ with the rise of hydrogen backpressure and a consequent increase of MgB_2_ was found. In some works [50,51], the formation of gaseous B_2_H_6_ was proposed as an intermediate of the LiBH_4_ decomposition. However, investigations on destabilized hydride systems suggested that the formation of gaseous B_2_H_6_ is kinetically precluded by the hydrogen backpressure [49,53,54]. Yan et al. did not find any formation of Li_2_B_12_H_12_ at 20 bar of hydrogen backpressure, leading to nearly one-step dehydrogenation [38]. Kim et al. [49] found no formation of the intermediate phase of Li_2_B_12_H_12_ during the dehydrogenation of 2LiBH_4_:1MgH_2_ at 450 °C and 10 bar of hydrogen backpressure. At 450 °C, it was reported that the equilibrium pressure of the LiBH_4_ decomposition into Li_2_B_12_H_12_ + LiH + H_2_ is 9 bar [55]; thus this equilibrium pressure is below the 10 bar of hydrogen backpressure, suppressing the formation of Li_2_B_12_H_12_ and resulting in an improvement of the dehydrogenation kinetics.

Cova et al. [56] performed a thorough analysis of the reaction paths of the 2LiBH_4_:1MgH_2_ under equilibrium conditions, mainly for the hydrogenation process. In the case of the dehydrogenation process in equilibrium conditions between 340 °C and 450 °C, they noticed that the inhomogeneous distribution between LiBH_4_ and MgH_2_ particles in the powder material results in different reaction completion times and hydrogen capacities for the first PCI (pressure–composition isotherm). Two dehydrogenation plateaus were evidenced: the higher and lower plateau for the decomposition of MgH_2_ and LiBH_4_, respectively, following the two-step reaction observed under dynamic conditions [33,34,35,55]. Additionally, cycling the Ni-added 2LiBH_4_:1MgH_2_ under dynamic conditions (425 °C and 6 bar H_2_) showed that the Li_2_B_12_H_12_ appears after the first dehydrogenation. It was suggested that this stable compound hinders the full reversibility, and thus it is responsible for the loss of hydrogen capacity [56]. Puszkiel et al. [39] and Jepsen et al. [40] also studied the dehydrogenation behavior of Li-RHC under equilibrium conditions by PCI curve measurements with different additives, i.e., 2LiH + MgB_2_ + 5 mol% TiO_2_ [39] and 2LiH + MgB_2_ + 5 mol% TiCl_3_ [40]. The two-step reaction was also verified in both cases, providing enthalpy values in excellent agreement with the ones for MgH_2_ for the higher plateau (~76 kJ mol^−1^ H_2_ [39] and ~73 kJ mol^−1^ H_2_ [40]), and enthalpy values in the order of 50–60 kJ mol^−1^ H_2_ for the second plateau ascribed to the dehydrogenation of LiBH_4_ and formation of MgB_2_ + LiH (~61 kJ mol^−1^ H_2_ [39] and ~53 kJ mol^−1^ H_2_ [40]). In the case of the hydrogenation process under equilibrium conditions, Cova et al. [56] identified two temperature regions below and above 413 °C. On the one hand, above 413 °C, two plateaus were noticed: the first (lower) one corresponding to the formation of LiBH_4_ from MgB_2_ and LiH and the second (higher) belonging to the hydrogenation of Mg. Noteworthy, the enthalpy value for the formation of LiBH_4_ amounted to ~41 kJ mol^−1^ H_2_, in accordance with the 40.5 kJ mol^−1^ H_2_ reported by Vajo et al. [29], while the obtained enthalpy value for the hydrogenation of Mg (higher plateau) was ~76 kJ mol^−1^ H_2_. On the other hand, below 413 °C, just one hydrogenation plateau was measured, which corresponds to the simultaneous hydrogenation of LiBH_4_ and MgH_2_.

Table 1 summarizes the proposed reactions pathways for Li-RHC with different additives and at different temperature and pressure conditions. Based on this analysis, it is clear that the mutual destabilization effect between LiBH_4_ and MgH_2_ only occurs upon hydrogenation, but at relatively low temperatures (<413 °C) under equilibrium conditions. For the hydrogenation process carried out under dynamic conditions, the applied temperatures were usually in the range between 300 °C and 400 °C [33,34,35,36,37,38,56,57,58,59,60,61,62,63,64,65,66,67]; hence, the mutual destabilization effect was verified by a one-step curve of hydrogen uptake against time. However, for the dehydrogenation, the thermodynamics limits the behavior of Li-RHC to two main reaction steps, losing the benefit of the destabilization effect. Under dynamic conditions, the dehydrogenation process of Li-RHC is hugely dependent on the hydrogen backpressure owing to the formation of the MgB_2_, which makes possible the reversibility of the system at temperature and pressure conditions milder than those needed for the formation of LiBH_4_ from free B and LiH.

Pristine Li-RHC presented sluggish kinetic behavior, requiring long hours for the hydrogen uptake and, principally, for the hydrogen release. Even for the understanding of the reaction pathway of pristine Li-RHC, different additives were employed to accelerate its kinetic behavior, as shown in Table 1. The most applied approach to improve the kinetic behavior of the 2LiBH_4_ + MgH_2_ system was the addition of transition metal (TM) and transition metal compounds (TMC) via mechanical milling [68]. Several works were published about the improvement of the kinetic behavior, and also cycling stability of TM- and TMC-added Li-RHC [56,57,58,59,60,61,62,63,64,65,66,67]. In 2010, Bösenberg et al. [57] studied the effects of TMC on the kinetic behavior of Li-RHC and proposed global reaction rate mechanisms for the absorption and mainly desorption of hydrogen. Applying gas–solid models, it was found that for all TMC additives, the rate-limiting of the hydrogenation is related to the interface velocity of the MgB_2_ decomposition described by a contracting-volume model. For dehydrogenation, kinetic behavior is the interface-controlled growth of MgB_2_ in one dimension. It was demonstrated that the addition of TMC leads to the formation of nanostructured transition metal boride (TMB) species with a similar crystal structure to MgB_2_. Therefore, it was proposed that nanostructured TMB species can act as heterogeneous nucleation sites for MgB_2_, thus improving the dehydrogenation rate of the slowest step, i.e., the decomposition of LiBH_4_ (second step), since the decomposition of MgH_2_ is quite fast. Based on this concept, either TMBs were added to Li-RHC or TM, and TMC were used as sources to form in situ TMB through the interaction with the Li-RHC [40,56,57,58,59,60,61,62,63,64,65,66,67]. Puszkiel et al. [39,69] proposed another mechanism to explain the effect of a specific TMC on the kinetic behavior of Li-RHC. Adding TiO_2_ led to the in situ formation of core-shell Li_x_TiO_y_ nanoparticles. It was found that these nanostructured core-shell Li_x_TiO_y_ species act as Li^+^ pumps, accelerating both the hydrogenation and dehydrogenation process. Furthermore, a novel kinetic model for the two-step dehydrogenation reaction was developed [39], which can be applied to the Li-RHC independently to the kind of used additive [65,67].

Table 2 provides a summary of some representative additives, describing hydrogenation and dehydrogenation conditions, capacities, and times as well as cycling stability (when it is available). As seen, the hydrogen capacities are between ~6 wt.% and ~10 wt.%, the temperature range is from 350 °C to 400 °C, the hydrogenation and dehydrogenation time range from 5 min to 4 h and from 30 min to 5 h, respectively, and the reported cycling covers up to 25 cycles with a measurable loss of capacity. Even though the hydrogen storage properties of Li-RHC were markedly improved, it is still a major constraint for a practical application that temperatures over 350 °C are required.

## 3. Metal and Metal Hydride Added LiBH_4_

After discussing the characteristics of the destabilization caused by mixing LiBH_4_ with MgH_2_ (see Section 2), the destabilization concept emerged as a promising alternative to access the high H_2_ content of strongly bound hydrides. Then, due to a large amount of potential destabilizing agents, some theoretical analyses were performed in order to evaluate thermodynamically the possibility of destabilizing this borohydride by its reaction with light elements or other hydrides. The equilibrium decomposition temperatures under different hydrogen pressures and the equilibrium phases were predicted for different reaction systems with a hydrogen content higher than 5 wt.% and including at least one reversible hydride. In particular, Cho et al.’s calculations considering the use of Al revealed a decrease in the decomposition temperature at 1 bar H_2_ from 403 °C for pure LiBH_4_ to 188 °C for the destabilized LiBH_4_ + Al system [70]. Moreover, they showed that the LiBH_4_ + AlH_3_ system was irreversible due to the high stability of AlH_3_, which is practically impossible to prepare by direct hydrogenation of Al as the required hydrogen pressure is of the order of 10^4^ bar. With the same aim, Siegel and co-workers used first-principles calculations to evaluate the thermodynamic properties of a series of reactions aimed at destabilizing lithium and calcium borohydrides by mixing with various elemental metals and/or their binary hydrides [71]. As can be seen in Table 3, strongly destabilized mixtures involving TiH_2_ can be highlighted, as well as moderately destabilized systems involving ScH_2_ and Cr which were found to possess thermodynamic properties that enable ambient hydrogen storage.

Motivated by these thermodynamic predictions, some experimental studies were carried out, screening many possible combinations [72,73]. Yang et al. [72] investigated several of the promising predicted reactions, specifically destabilizing LiBH_4_ both with metals (Al, Mg, Ti, V, Cr, Sc) and with metal hydrides (MgH_2_, TiH_2_, and CaH_2_), whereas Au and co-workers [73] evaluated the effectiveness of various metals (Mg, Ni, Al, Ca, In), metal hydrides (MgH_2_, NaH, CaH_2_) and metal chlorides (MgCl_2_, TiCl_3_) as destabilizing agents. In contrast to the theoretical calculations, the TPD-MS screening of LiBH_4_ modified samples showed no hydrogen release events at temperatures below 300 °C [72,73]. Although certain additives (NaH, Ni, Ca) showed a negative effect due to the formation of more stable metal borohydrides [73], for all the other combinations the peak corresponding to the H_2_ desorption temperature was shifted to lower values relative to that of pure LiBH_4_, suggesting a weak thermodynamic destabilization and/or kinetic enhancement [72,73]. Isothermal desorption showed slow kinetic behavior for all mixtures, unable to achieve full desorption even after 100 h at 400 °C (see Table 3) [72].

The formation of stable metal borides is expected as it represents the feasible thermodynamic pathway [71]. Despite this, the experimental evidence shows that metal or metal hydrides can be found as products [72]. For samples LiBH_4_-M (M = Cr, V, Sc, Ti) and LiBH_4_-MH_2_ (MH_2_ = TiH_2_), the observed desorption corresponds to the LiBH_4_ decomposition. In these cases, the M or MH_2_ starting material remains either unreacted (for Cr and TiH_2_) or, when thermodynamically favored, forms a stable hydride (for V, Sc, and Ti). Kinetic limitations can be related to reduced atomic diffusion of the additive metal, hydride, or boride species at temperatures at which the desorption reaction occurs. These additives do not destabilize LiBH_4_ thermodynamically, but they act as catalysts [72]. On the other hand, for samples LiBH_4_-M (M = Al, Mg) and LiBH_4_-MH_2_ (MH_2_ = CaH_2_, MgH_2_), the expected metal boride phases are formed (with high dependence from temperature and pressure conditions), validating the thermodynamically predicted reaction products [72,73]. In this section, the use of Mg and its hydride as destabilizing agents are not included as it has been presented previously, but the Al and CaH_2_ cases, which constitute light destabilization agents, are here analyzed.

**Figure 3:**
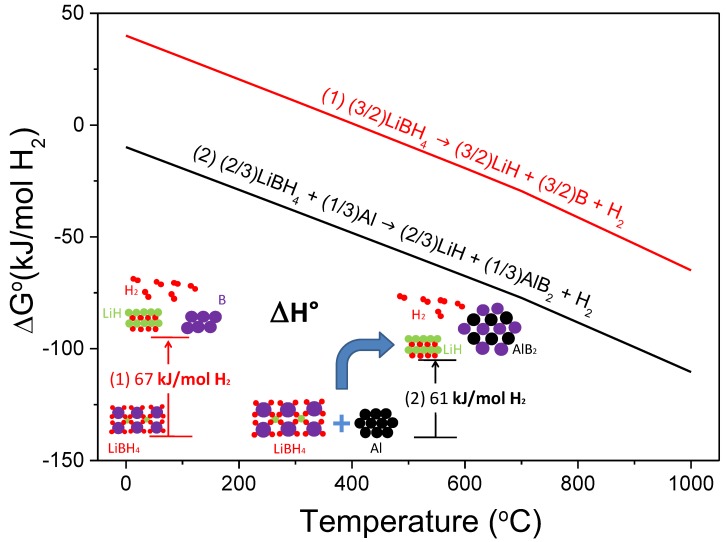
Free energy per mol of H_2_ as a function of temperature and standard enthalpy of reaction of the hydride system 2LiBH_4_ + Al.

Al-doped LiBH_4_ has attracted considerable attention for its enhanced performance of reversible hydrogen storage. This system has a theoretical capacity of 8.6 wt.%, and it was proved to be reversible with variated conditions for rehydrogenation (see Table 4). Figure 3 shows the free energy per mol of H_2_ and the standard enthalpy of reaction per mol of H_2_ resulting from the destabilization effect of Al on LiBH_4_ according to Reaction (2), calculated with HSC software [28].

Interestingly, the value of the enthalpy for the dehydrogenation (Reaction (2)) has been controversially debated. Due to the uncertainty in the stability of the involved products, especially regarding AlB_2_ which calculated formation enthalpy varies from 23 to 151 kJ mol^−1^, different published reaction enthalpies can be found: ΔH = 18.8 kJ mol^−1^ H_2_ [71,74], ΔH = 44.2 kJ mol^−1^ H_2_ [70], ΔH = 59.3 kJ mol^−1^ H_2_ [75]. Nevertheless, experimental analyses showed that the Al-destabilizing effect on LiBH_4_ is smaller than the calculated [75]. The composite 2LiBH_4_–Al has been suggested to release hydrogen in two steps represented by Reactions (2) and (3):

2LiBH_4(l)_ + Al_(s)_ → AlB_2(s)_ + 2LiH_(s)_ + 3H_2(s)_ (2)

2LiH_(s)_ + 2Al_(s)_ → 2LiAl_(s)_ + H_2(g)_ (3)

However, as the pressure–temperature conditions are conducive for both reactions to occur on the basis of thermodynamics, the decomposition of LiBH_4_ (Reaction (4)) occurs along with the formation of AlB_2_ (Reaction (2)) and it was proved that by increasing the desorption backpressure, a relative enhancement in the contribution of the desired boride-forming reaction was achieved [72,76,77]. Moreover, the occurrence of Reaction (5) explains that an autocatalysis reaction dominates the process: the products AlB_2_ and Al serve as reagents for the decomposition of LiBH_4_ [76].

2LiBH_4(l)_ → 2LiH_(s)_ + 2B_(s)_ + 3H_2(s)_ (4)

2LiBH_4(s)_ + AlB_2(s)_ → 2LiH_(s)_ + Al_(s)_ + 4B_(s)_ + 3H_2(g)_ (5)

The experimental H_2_ capacities reported for the first desorption are in the range of 5.1–8.4 wt.%, this is 60–97% of the theoretical H_2_ content. Unfortunately, hydrogen release and uptake for LiBH_4_−Al reveals a significant degradation in the H_2_ storage capacity [72,74,77,78] (See Table 4): the capacity was proved to be reduced by half in four cycles [74], or even degraded to ~15% of the theoretically available H_2_ content in 10 cycles [77]. The capacity loss may be due to the combination of several factors [74,77,78,79,80,81]: (1) the incomplete reaction between LiBH_4_ and Al; (2) segregation of B as amorphous clusters, not participating in the formation of AlB_2_; (3) formation of B_2_H_6_ at temperatures below 300 °C; (4) presence of trace amounts of BH_3_ and H_2_O from the decomposition of commercial LiBH_4_; (5) formation of Li_2_B_x_H_x_-type species such as Li_2_B_12_H_12_ or boron phase that do not participate in the reversible reaction; (6) available free Al decrease due to the formation of a passivation layer composed of reaction products on its surface. Recently, it was shown that the extent of the dehydrogenation reaction greatly depends on the precipitation and growth of reaction products (LiH, AlB_2_, and LiAl) on the Al surface. Then, a passivation shell formed by these products may be the kinetic barrier to the dehydrogenation of the Al-doped LiBH_4_ composite [80,81].

Based on the promising thermodynamic calculations, the incorporation of calcium-based compounds for LiBH_4_ destabilization has been widely explored. Although the addition of metallic calcium is not sufficient to destabilize LiBH_4_ because the metal is covered by a CaO layer preventing the Ca from interacting with LiBH_4_ [73], it has been demonstrated that CaH_2_ promotes hydrogen liberation from LiBH_4_. The coupled 6LiBH_4_/CaH_2_ system has a theoretical hydrogen capacity of 11.7 wt.% and constitutes an example of a LiBH_4_-destabilization reaction involving a MH_2_ that occurs at conditions below the decomposition of the metal hydride itself, which suggests that low-decomposition-temperature hydrides are not an essential component for LiBH_4_ destabilization [72]. The system has been studied with the addition of several dopants (TiF_3_, TiO_2_, TiCl_3_, V_2_O_5_, NbF_5_), which showed having a small effect on the desorption temperature. The catalyzed systems released 9–9.4 wt.% reversibly at 400–450 °C via the boride forming reaction in a single step, Reaction (6) [82,83,84,85,86]:

6LiBH_4(l)_ + CaH_2(s)_ ⇆ 6LiH_(s)_ + CaB_6(s)_ + 10H_2(g)_ (6)

Despite the fact that some studies attribute the failure in achieving reversibility of the system without any dopant to the lack of mobility of the metal boride product phase [42,72], the presence of a catalytic additive seems to be critical in lowering the kinetic barrier of the hydrogenation reaction, allowing rehydrogenation at 400–500 °C and 80–100 bar [82,83,84]. The analysis of successive sorption–desorption cycles showed that the NbF_5_-doped system maintains a reversible hydrogen storage capacity of about 6 wt.% at 450 °C after a slight degradation between the 1st and 5th cycle, suggesting that the additive improves the cycle properties by retarding microstructural coarsening [85]. Kinetic modeling measurements demonstrated that the reaction is controlled by mixed processes [87]: reaction at the phase boundary controls desorption rates initially, whereas diffusion [84] does it in later stages. As the accuracy of thermodynamic data of LiBH_4_ and CaB_6_ is questionable, different calculations of the reaction enthalpy have been published [82,84,88] (see Table 5). Consistently, an experimental value of ΔH = 56.5 kJ mol^−1^ H_2_ was obtained, which means that the equilibrium temperature under 1 bar of H_2_ is 309 °C [84].

Summarizing, theoretical calculations showed the potential of the LiBH_4_ destabilization by chemical reaction approach as a possible solution for on-board applications. Experimental investigations demonstrated that whereas certain additives showed a negative effect due to the formation of more stable metal borohydrides (NaH, Ni, Ca) or act only as catalyst (M = Cr, V, Sc, Ti, In or MH_2_ = TiH_2_), other additives (M = Mg, Al or MH_2_ = MgH_2_, CaH_2_) do destabilize LiBH_4_ thermodynamically following the expected formation of a metal boride product. Though thermodynamically promising, kinetic energy barriers have to be overcome. The destabilized lithium borohydrides with metals or hydrides are reversible in limited cycles with slow reaction kinetics, and the required rehydrogenation temperature and pressure are still elevated.

## 4. Destabilization of LiBH_4_ by Rare Earth (RE) Metal Hydrides

Among the possible metal hydrides tested to destabilize LiBH_4_, the rare earth (RE) metal hydrides constitute an attractive group of compounds due to the improvements in the theoretical thermodynamic parameters respect to pure LiBH_4_ decomposition as well as in the LiBH_4_ dehydrogenation kinetics [54,83,89,90,91,92,93,94,95,96,97,98,99]. Considering that several RE hydrides are not commercially available, three different approaches have been used to produce these hydrides (See Table 6) and to form in the following step the based-LiBH_4_ destabilized composites. In the first synthesis procedure, pure RE metal reacts with hydrogen gas at a defined temperature and pressure in a closed-reactor, according to the thermodynamic information available [100]. The second synthesis method promotes the reaction between RECl_3_ and LiH by mechanochemical activation, forming REH_3_ together with LiCl as a by-product. The third strategy involves the in situ formation of RE hydrides during decomposition of some RE borohydrides. These RE borohydrides can be obtained by milling of LiBH_4_ and RE halides, mainly the LiBH_4_-RECl_3_ or LiBH_4_-RECl_3_-LiH mixtures, where LiCl is formed as a by-product. In some cases, the milling induces partial halide substitution in the RE borohydride [101,102]. Afterward, for the case of the three synthesis procedures, the as-synthesized RE hydride and LiBH_4_ powders are mixed using ball milling, obtaining new destabilized composites with promising properties.

The first investigation about LiBH_4_ destabilization by REH_2_ was done in 2008 using ScH_2_ [89]. Thermodynamic calculations predicted for the LiBH_4_-ScH_2_ system a reaction enthalpy of 34 kJ mol^−1^, with a hydrogen release of 8.9 wt.% upon completion, and decomposition temperature of 57 °C at 1 bar [89]. However, experimental results showed that only 4.5 wt.% of hydrogen was released after 20 h at 450 °C. The evidence suggests that ScH_2_ does not participate in the LiBH_4_ decomposition, probably due to its high stability: decomposition of ScH_2_ does not occur until 900 °C compared with 275 °C for MgH_2_. The use of nanostructured ScH_2_ was expected to promote the interaction between the hydrides by creating excess surface energies and excess grain boundary enthalpies. However, above the melting point of LiBH_4_, the nanostructured mixture segregates back into distinct hydride phases, without evidence of ScB_2_ formation. The authors confirm that ScH_2_ is not effective in destabilizing LiBH_4_.

**Figure 4:**
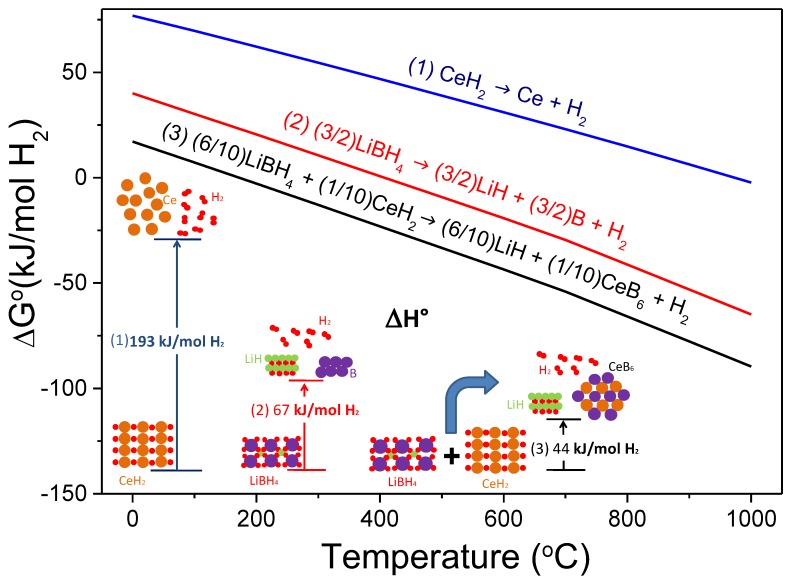
Free energy per mol of H_2_ as a function of temperature and standard enthalpy of reaction of the hydride system 6LiBH_4_ + CeH_2_.

In the same year, Jin et al. were pioneers in successful destabilizing LiBH_4_ with CeH_2_ [83]. They assessed the theoretical enthalpy change of both LiBH_4_-Ce and LiBH_4_-CeH_2_ systems (27.6 and 44.1 kJ mol^−1^, respectively) and predicted a significant lower dissociation temperature than the individual hydrides (171 °C versus >350 °C [1]), according to Reaction (7). Experimental studies confirmed that the 6LiBH_4_-CeH_2_ composite catalyzed by 0.2TiCl_3_ release hydrogen at a temperature lower than LiBH_4_ melting forming CeB_6_ and LiH as solid products, in agreement with thermodynamic calculations. Figure 4 exhibits the free energy per mol of H_2_ and the standard enthalpy of reaction per mol of H_2_ resulting from the destabilized 6LiBH_4_ + CeH_2_ system according to Reaction (7), calculated with HSC software [28].

6LiBH_4(s)_ + CeH_2(s)_ → 6LiH_(s)_ + CeB_6(s)_ + 10H_2(g)_ (7)

The system showed good reversibility after rehydrogenation at 350 °C under 100 bar of hydrogen for 20 h, maintaining 6 wt.% of hydrogen capacity respect the nominal 7.4 wt.% without catalyst [83]. However, the theoretical enthalpy value calculated was lower than that ΔH = 58 ± 3 kJ mol^−1^ obtained by dynamic pressure-composition isotherms, implying a higher decomposition temperature of 240 ± 32 °C at 1 bar [90]. Different factors, such as kinetic restrictions and deviations from the equilibrium conditions during the experimental determination of the equilibrium pressure, influence the obtained results.

Motivated by the positive effect of the hydrogen back pressure on the formation of MgB_2_ in the LiBH_4_-MgH_2_ decomposition [29,34,43], Shim et al. studied whether it was a general trend for the destabilization of LiBH_4_ based-systems [91]. Investigations on the LiBH_4_-CeH_2_, LiBH_4_-YH_3_, and LiBH_4_-CaH_2_ composites demonstrate an enhancing effect of hydrogen back pressure on the LiBH_4_ destabilization reaction by the formation of metal borides CeB_6_, YB_4_, and CaB_6_, respectively. The experimental evidence suggested that the enhancing effect of hydrogen backpressure is general for all destabilized LiBH_4_ composites. In particular, the new explored 4LiBH_4_-YH_3_ composite released 7.0 wt.% of hydrogen (theoretical 8.4 wt.%) at 350 °C under 0.5 MPa of hydrogen backpressure through the formation of YB_4_ and LiH as shown in Reaction (8):

4LiBH_4(s)_ + YH_3(s)_ → 4LiH_(s)_ + YB_4(s)_ + 7.5H_2(g)_ (8)

About 70% of reversibility was confirmed for this system under mild conditions (350 °C and 9.0 MPa for 24 h), without using a catalyst. As an interesting result, argon back pressure showed a similar effect that hydrogen on the dehydrogenation due to the suppression of diborane formation [54]. Through thermodynamic experimental and theoretical studies of 4LiBH_4_:YH_3_ composite, the reaction enthalpy was estimated being 52 kJ mol^−1^ and 48 kJ mol^−1^, respectively [92]. The dehydrogenation temperature calculated from the experimental data was 232 °C at 1 bar, which is higher than the calculated value (180 °C) but significantly lower than that of LiBH_4_ [29].

The first investigation about the destabilization of LiBH_4_ by in situ formation of RE hydrides was reported by Gennari et al. [93]. The 6LiBH_4_-RECl_3_ mixture (RE = Ce, Gd) was milled, and new composites containing LiBH_4_, RE borohydride, and LiCl phases were formed. These composites display superior hydrogen storage properties than LiBH_4_. Hydrogen release starts at 200 °C due to the RE borohydride decomposition forming in situ REH_2+x_, which promotes LiBH_4_ decomposition, with additional hydrogen release up to 400 °C (80% of theoretical value). The formation of CeB_6_ and GdB_4_ in the dehydrogenated state was demonstrated for each system, simultaneously with LiH and LiCl as secondary phases. In order to promote the reversibility of CeB_6_ formed in the dehydrogenation reaction, the addition of LiH to the as-milled LiBH_4_-CeCl_3_ (6:1) composites was tested. The presence of 3LiH in the initial 6LiBH_4_-CeCl_3_ mixture allowed the formation of Ce hydride by direct reaction between CeCl_3_ and LiH, avoiding the formation of Ce borohydride, following Reaction (9) [93]. The new destabilized system showed 80% of reversibility using mild conditions (400 °C and 6.0 MPa of hydrogen during 2 h) and without catalysts.

6LiBH_4(s)_ + CeCl_3(s)_ + 3 LiH_(s)_ → 6LiH_(s)_ + CeB_6(s)_ + 3LiCl_(s)_ + 10.5H_2(g)_ (9)

The relevance of the nanostructure of the LiBH_4_-CeH_2+x_ composite on the hydrogen storage properties was showed in [94]. Superior sorption rates and hydrogen storage reversibility were reached when CeH_2+x_ was formed in situ due to its nanostructured features. The addition of ZrCl_4_ forms Zr(BH_4_)_4_ in situ [108] and produces nanometric ZrB_2_, which increases the nucleation sites improving the dehydrogenation rate. Gennari reported for the first time the destabilized LiBH_4_-REH_2_ (RE = Ce, La) composite, where LaH_2+x_ was produced by milling of the LaCl_3_ and LiH [86]. The onset temperature of hydrogen release was 260 °C, similar to LiBH_4_-CeH_2+x_ composite. However, the LiBH_4_-LaH_2+x_ system possesses inferior hydrogen storage reversibility than LiBH_4_-CeH_2+x_.

Following this, Gennari explored the in situ formation of YH_2+x_ by the milling of 4LiBH_4_-YCl_3_ plus 3LiH. The dehydrogenation behavior was improved by the reduction of the decomposition temperature of LiBH_4_, showing 80% of hydrogen storage reversibility [95]. The hydrogen backpressure affects LiBH_4_ dehydrogenation: its increase favors the YB_4_ formation and suppresses the formation of diborane. In contrast, a reduction of the hydrogen backpressure induces the formation of Li_2_B_12_H_12_, which restricts posterior rehydrogenation. In general, the thermodynamic destabilization of LiBH_4_-RE hydrides was shown; however, the kinetic enhancement obtained in the first dehydrogenation was progressively lost upon cycling. Different factors, such as the deterioration of the nanostructure/microstructure of the composite, the formation of Li_2_B_12_H_12_, and the high kinetic barrier for the nucleation of the new phase, are among some of the possible drawbacks for the full reversibility of the system.

Based on a previous investigation on 4LiBH_4_-YH_3_ and the higher hydrogen desorption temperatures induced by an incubation period as a consequence of a loss of nanostructure [54,91,94], Cai et al. explored the destabilized LiBH_4_-NdH_2+x_ composite and analyzed the role of the NdH_2+x_ microstructure on hydrogenation–dehydrogenation cycles [96]. The calculated dehydrogenation enthalpy changes through the formation of NdB_4_ amounted to 64 kJ mol^−1^ and the theoretical hydrogen storage capacity of 6.0 wt.%. Experimentally it was observed that hydrogen was quickly released at 370 °C in 1.5 h due to the nano-sized nature of NdH_2+x_ [96]. Good reversibility of 6.0 wt.% and 5.2 wt.% of H_2_ was observed in 3 h and 2 h for the second and third cycles, respectively. However, in subsequent re-/dehydrogenation cycles, the NdH_2+x_ particles coarsen, hence the loss of nanostructure restricts its interaction with LiBH_4_ diminishing the destabilization effect. The consequences are higher temperatures for dehydrogenation and more inferior kinetic behavior for dehydrogenation. The authors proposed that reducing and stabilizing the particle size of NdH_2+x_ would lead to a better-destabilized system for hydrogen storage applications.

A systematic investigation on the thermal behavior of as-milled 6LiBH_4_-RECl_3_ (RE = La, Ce, Pr, Nd, Sm, Eu, Gd, Tb, Er, Yb and Lu) mixtures was developed by Olson et al. [97]. In this study, only the rehydrogenation of the 6LiBH_4_-RECl_3_ (RE = La, Er) systems was explored. For the milled 6LiBH_4_-RECl_3_ mixture where RE = La, Ce, Pr and Nd, dehydrogenation starts below 200 °C and proceeds up to 350 °C, involving complex interactions as the temperature increases, inducing Cl-substitution in LiBH_4_, undergoing partial decomposition of RE borohydride, and leading to lower dehydrogenation temperatures than pure LiBH_4_ as a result of the interaction between LiBH_4_ and RE hydrides [102]. No emission of diborane or other borane species was detected. In the case of the as-milled 6LiBH_4_-RECl_3_ composite (RE = Gd, Tb, Er, and Lu), the first RE-borohydride decomposes around 250 °C releasing hydrogen and then leading to the dehydrogenation of LiBH_4_. In contrast, the as-milled 6LiBH_4_-RECl_3_ containing Sm, Eu, and Yb displayed a different thermal trend, showing just one main mass loss event at temperatures lower than 200 °C. TG curves exhibited significant weight loss at 180 °C, 150 °C, and 100 °C for the Sm, Yb, and Eu composites, respectively. This behavior was associated with the reduction from trivalent to the divalent state of the RE metal with simultaneous diborane release. Partial rehydrogenation (18% and 25%) was observed for RE = La, Er at 300 °C, and 415 °C, respectively, using 10 MPa of hydrogen, after complete dehydrogenation under vacuum.

In order to promote the reversibility of 6LiBH_4_-RECl_3_ (RE = La, Er), Frommen et al. studied the addition of 3LiH on the hydrogen storage properties [98]. The destabilized composite containing LaH_2+x_ showed limited rehydrogenation capacity (˂20%) at 340 °C under 10 MPa hydrogen pressure, probably due to hydrogen uptake by some amorphous phases. In the case of the composite containing ErH_2+x_, desorption against 0.5 MPa of backpressure favors 80% of reversibility as compared to vacuum (60%) via the formation of ErB_4_. Rehydrogenation at 340 °C and 10 MPa show the formation of ErH_3_ and LiBH_4_ at mild conditions compared to pure LiBH_4_. Additional studies on LiBH_4_-Er(BH_4_)_3_-LiH (3:1:3) composite, using Er borohydride free of LiCl, showed a hydrogen release of 4.2 wt.%, 3.7 wt.% and 3.5 wt.% after consecutive cycles (400 °C, 5–10 bar of H_2_) [99]. Rehydrogenation was performed at 340 °C and 100 bar of H_2_, reaching a reversible hydrogenation capacity of about 80–85%. The hydrogen storage properties obtained from Er borohydride LiCl-free were similar to a 6LiBH_4_-ErCl_3_-3LiH composite mixture obtained by milling [98].

Table 7 summarizes the main hydrogen storage properties of the selected composites obtained from the LiBH_4_-REH_2+x_, LiBH_4_-RECl_3_, or LiBH_4_-RECl_3_-LiH mixtures. As a general behavior, as-synthesized RE hydrides or RE hydrides formed from the decomposition of RE borohydrides are active additives to destabilize LiBH_4_. In the first case, RE hydrides promote the destabilization of LiBH_4_ by direct formation of RE boride and LiH, with enhanced reversibility under mild conditions (RE = Ce, Y). The influence of nanostructure as well as of the hydrogen backpressure or the use of catalysts were tested. In the case of RE hydrides produced from RE borohydrides, the decomposition is a multistep process through intermediate phases until the formation of RE borides and RE hydrides. Hydrogen release occurs at the same temperature as the onset temperature of RE borohydride decomposition, with the consequent RE hydrides formation (RE = Ce, Y, La, Gd, Er). LiBH_4_ releases hydrogen at lower temperatures than as-milled LiBH_4_ due to the interaction with RE hydrides. The addition of 3LiH in the initial mixture promotes the hydrogen storage reversibility (RE = Er).

## 5. Nanoconfinement of LiBH_4_

### 5.1. Outline

Nanoconfinement promotes hydrogen exchange in hydrides submitted to the physical constraints of an inert matrix. The crystallinity of the hydride is lost at the vicinity of the interface, and melting is prompted while the diffusion path of the reactive species is limited to the dimensions of the cavities. The sides of the matrix limit particle growth and phase segregation, improving reversibility. These effects are appealing for LiBH_4_ as (i) it melts before it decomposes, (ii) it needs active species to migrate at the surface of the material, (iii) its reversibility suffers from numerous by-products due to a variety of chemical paths. Several excellent reviews devoted to complex hydrides treat the specific subject of nanoconfinement [4,13,14,21,24,25]. Here, we propose to focus specifically on the effects of nanoconfinement over LiBH_4_ in order to compare the strategies proposed to understand and enhance its properties.

### 5.2. Techniques

Differential scanning calorimetry (DSC) of just-impregnated samples depicts the crystalline state of the nanoconfined hydride associated with its temperatures of transition, melting, and decomposition; thus, it correlates the structure of the material with its efficiency. Coupled with XRD, those techniques indicate if the hydride is within the matrix through the smoothing of their signal, as nanoconfined hydrides lack of long-range order. These methods are highly informative as long as the effect of nanoconfinement is moderate. Once the pores are very small (<4 nm), these techniques turn solely qualitative (is the hydride confined or not) as any signal disappears because the material turns wholly amorphous [109]. At this point, the team must rely mostly on FTIR to ensure that LiBH_4_ is present. The orthorhombic to hexagonal transition peak (between 100 °C and 120 °C) can be particularly illustrative of the enhancement originated from nanoconfinement, and Suwarno et al. dedicated a full article to this specific transition [110]. It is also very instructive to focus on its splitting behavior: with pores of roughly 10 nm, one peak depends on the size of the pores (the smaller the pores, the lower its temperature) while a second stays close to 120 °C. As the later value is very similar to the temperature observed for bulk LiBH_4_, this peak was commonly attributed to LiBH_4_ that remained outside of the pores. Yet, some experimental observations somewhat disagreed with this assumption: (i) that peak is particularly marked for bigger pores (which arguably should be easier to fill); (ii) that peak is present even at lower pore filling; (iii) if bulk LiBH_4_ was present, its melting peak should be clearly observed, which is not necessarily the case. We proposed that these peaks are the inner and outer sphere of nanoconfined LiBH_4_, the outer suffering the highest effect of nanoconfinement while the inner sphere acts more likely to the bulk, in accordance with the work of Suwarno et al. [111]. Additionally, they highlighted by DSC that the deviation of the nanoconfined material melting point concerning its bulk is proportional to the inverse of the pore radius of the scaffold (ΔT_fus_ = f(1/r_pore_)).

Adsorption/desorption isotherms (commonly under N_2_) allow determining how much hydride can be infiltrated within a matrix and offer an estimation of the average pore-size, which affects the efficiency of the nanoconfinement, particularly below 4 nm [109,112]. Hitherto, some authors obtained excellent results with not so small pore sizes [113]. Once the material was infiltrated, the remaining pore volume can be superior or inferior to the expected one. Both discrepancies can be related respectively to (i) some hydride remaining outside of the matrix, (ii) the pores being clogged, (iii) each phenomenon occurring simultaneously. Small-angle neutron scattering (SANS) is a complementary technique that is not affected by clogging and permit to attribute each such discrepancy [114]. As such, wetting is an essential property for the nanoconfinement of hydrides, particularly to reach higher filling values in smaller pores; so far, the insights are very scarce to date on this topic [115,116].

Suwarno et al. employed quasi elastic neutron scattering (QENS) to verify the effect of the chemical interaction between the matrix and the hydride over the temperature of desorption [100]. They observed that the effects of nanoconfinement were more pronounced for silica scaffolds than for carbon ones. In the vicinity of the pore walls, an “active” layer of LiBH_4_ was observed, thicker for SiO_2_ matrices (1.94 nm vs. 1.41 nm for C matrices). QENS showed that the fraction of LiBH_4_ with high hydrogen mobility was more significant in silica. Verdal et al. distinguished by QENS two populations of LiBH_4_ of distinct mobility and studied the effect of pore size and temperature over it [117].

In situ techniques would be very informative to understand the wetting, decomposition, and rehydrogenation processes. However, several such techniques are not adequate to investigate the whole nanoconfinement process. For instance, in situ X-Ray Diffraction (XRD) would provide a way to follow the impregnation process, hence to understand the phenomena that hinder the reversibility of the materials, but it suffers from the amorphous nature of the nanoconfined materials [114,118,119]. In situ Raman spectroscopy was performed by Miedema et al. [120]. While they could not follow the intermediates during the dehydrogenation, the technique allowed to compare as-prepared samples with rehydrogenated ones, and the presence of intercalated Li within the porous carbon was confirmed, as proposed later by House et al. [116]. They observed the formation of Li_2_B_12_H_12_ under mild conditions (1 bar H_2_, 350 °C) and confirmed the role of this species as an intermediary of rehydrogenation [121].

Shane et al. tried ions mobility by nuclear magnetic resonance (NMR) of nanoconfined LiBH_4_ [122]. As NMR is more susceptible to local structure than to long-range order, this technique very well suited to nanoconfined materials [123]. Using ^1^H, ^7^Li, and ^11^B NMR, they detected a broadening effect promoted by nanoconfinement as per DSC or XRD. Therefore, nanoconfined BH_4_^−^ and Li^+^ presented increased diffusional mobility in front of their bulk counterparts. Verkuijlen et al. compared C versus Si matrices and varied the weight loading of hydrides. Liu et al. combined NMR and QENS studies on LiBH_4_ confined within 4 nm pores to demonstrate that the ions are more mobile next to the interface than in the core, even for such small pores [124]. These contributions are paralleled only by simulations studies when it comes to an understanding of the mechanism underlying the effects of nanoconfinement [110,115].

### 5.3. Impregnation

Melt impregnation of a porous matrix at 300 °C under 60 bar H_2_ is the most common method to achieve the nanoconfinement of LiBH_4_. The method can slightly vary: Suwarno et al. claimed that repeating the impregnation allowed them to reduce the proportion of LiBH_4_ remaining outside of the pores [110]. Nevertheless, this point is scarcely discussed by the authors, while it is a relevant experimental parameter. House and Mason proposed that LiBH_4_ was not likely to impregnate a pure carbon matrix, but in its stead, the phenomenon was allowed by the serendipitous release of a small fraction of boron during phase transition/melting of the hydride, followed by its inclusion within the defects of the carbon matrix [115,116].

Wet impregnation is generally performed in a solution of dry THF but can lead to the formation of hard to remove etherates. Cahen et al. presented a sui generis wet impregnation method, with highly relevant crystallographic data. By using hindered ethers, they avoided the formation of stable complexes and confined LiBH_4_ within tiny pores (4 nm, plus a considerable amount of micropores, 1060 m^2^ g^−1^ specific surface area) [112]. The solute state of the hydride during the infiltration probably allows the smaller pores to be filled, thus the release of H_2_ at very low temperatures. Their material might present the best overall characteristics (6 wt.% H_2_, onset below 200 °C, 50% release at 235 °C), but the prolonged temperature increase is likely to be accountable for these values (as the material was allowed to dwell 2 h between each 50 °C). Regrettably, the authors stated that this material was not reversible, which they attributed to the pores being too big. However, Gross et al. obtained good reversibility with bigger pores [113]. More probably, the pores were clogged, or etherates are responsible for the enhanced first cycle and restrained reversibility, similarly to the effects described for silicates [125]. It should also be stated that rehydrogenation was conducted under a very moderate temperature (300 °C). Furthermore, during desorption, the pressure was loosely controlled between 0 and 1 bar to a very high final temperature (18 h to reach 500 °C). The combination of dwelling at high temperature and irregular pressure might very well promote the formation of non-reversible intermediary compounds (vide infra, Utke et al.). It should be noted that the authors hand-mixed their matrix with LiBH_4_, and surprisingly, no improvement was observed in comparison with the bulk material, even if at 300 °C one should have expected melt impregnation to occur.

### 5.4. Matrix

In their work, Fang et al. presented the effect of SWNT over LiBH_4_ [126]. While it was not an explicit goal of their work, it can be assumed that their results are among the first of nanoconfinement because (a) no effect was observed without milling (b) the effect decreased if milling was done for too long. Indeed, milling could open SWNT at first, and then the structure is gradually lost. Later they proposed to impregnate a matrix of activated carbon with LiBH_4_ by wet impregnation [127]. Similarly, Zhang et al. milled LiBH_4_ with a carbon scaffold, but this led to the partial destruction of the pores [128].

Gross et al. presented the prototypical nanoconfinement of LiBH_4_ within carbon aerogel scaffolds (CAS) [113]. Despite the relatively large pore size of their matrix, excellent results are described. It is essential to note that on DSC and XRD, the remain of crystal structure was present.

Ngene et al. proposed a very interesting silicate SBA-15 matrix [125]. Their results are excellent on the first step of hydrogen release, but then the reversibility suffered a lot, which was correlated to the degradation products of LiBH_4_ being reactive toward the Si matrix (while this matrix proved inert in front of LiBH_4_). They highlighted the specific role of Si in a simulation [110]. They also demonstrated the interest of doping a C-matrix with Ni and observed better reversibility of the system, even under moderate pressure (40 bar) and temperature (320 °C) for 2 h due to the formation of Ni_2_B [129,130]. To thwart phase segregation of LiH and B, they proposed to dope the C matrix with excess Li and observed improved reversibility [131].

Majzoub et al. contributed to the field by employing a matrix of narrow pore distribution, to discriminate pore size from pore filling [132]. Indeed, usually, CAS presents a broad pore distribution, so pore filling vary concomitantly with the size of the pores. They employed several nano porous carbon (NPC) of different pore sizes at distinct filling values to systematically evaluate the role of each factor [109]. Their material demonstrated good reversibility under reasonable conditions (60 Bar H_2_, 250 °C), yet suffered from low hydride weight capacity (20 wt.%). Importantly, they illustrated the effect of pore size over B_2_H_6_ release to narrow the factors limiting reversibility. They presented the pivotal role of the wetting of LiBH_4_ and LiH both experimentally and by simulations [115,116]. One limitation of nanoconfinement exposed by their work is that nanoconfined LiBH_4_ does not release H_2_ above a critical temperature (as should do a pure crystalline material). Indeed, while they used a tiny pore size and placed themselves above the temperature of the fastest dehydrogenation, they obtained a limited (7 wt.% of LiBH_4_) H_2_ desorption. Given the aim of their material was to use a narrow pore size distribution, the broadness of temperature during H_2_ release cannot be attributed to the broadness of pore size. They attributed some effects to LiBH_4_ remaining outside of the matrix, but DSC of their material might indicate some discrepancies (mostly the absence of melting peak from bulk). Later, they highlighted the gradient of ion mobility within the pore of small and controlled size [124]. More recently, this broadness of hydrogen release temperature was attributed to the core/shell structure of LiBH_4_ nanoparticles [109,133].

Despite that C-matrixes are the most commonly employed, the effect of graphene is mostly not studied. In our group, we presented a method to obtain graphene-doped aerogels in order to determine if the improvement of thermal conductivity might traduce in lower hydrogen release temperatures [111]. While the presence of graphene was not beneficial, it allowed us to obtain higher pore filling with comparable temperatures. The effect of pore size, pore-filling, and graphene was systematically studied following Yates plan in order to highlight interactions between these parameters. We observed an improved wetting of the matrix when graphene was present, in accordance with the insertion of boron within graphene defects as presented by Mason [115]. The solvothermal reaction of organometallic compounds (MgBu_2_, LiBu) under high hydrogen pressure (35, 50 bar) allowed Xia et al. to decorate graphene sheets with nanostructured hydrides [134,135]. 10 nm core-shell MgH_2_-LiBH_4_ nanoparticles and 2 nm thick LiBH_4_ layers were observed by TEM at the surface of the graphene support, even at very high weight loading (70–80%). This allowed both composites to exhibit excellent hydrogen capacity (9.1–12.8 wt.%), and the reactive hydride [134] displayed much-improved reversibility in front of the lone LiBH_4_ [135]. These materials present well-defined peaks by XRD, suggesting the hydride might not be nanoconfined. Thus, those excellent results should be attributed to the size of the particles, promoted and preserved by the high specific surface area of graphene. The authors also interestingly claimed the thermal conductivity and mechanical flexibility of graphene might play a role.

The effects of doping a C-matrix with heteroatoms are scarcely studied so far. Carr et al. presented how N-doping affects NaAlH_4_ [136], while we exposed the impact of this element over LiBH_4_ [133]. This N-doped material was decorated with metallic nanoparticles (Ni, Co, and their mixtures). Co-decorated matrices liberated hydrogen at lower temperatures, while Ni-decorated matrices presented enhanced reversibility [137]. Gosalawit-Utke et al. proposed an original PMMA–co–BM polymer matrix for the nanoconfinement of LiBH_4_ [138]. Most strikingly, their material presented a single exothermic peak at 158 °C by DSC, which was associated with both hydrogen release and thermal degradation of the matrix. Hydrogen was liberated at very low temperature (onset at 80 °C, main release at 105 °C). The drawback of this material was its very low weight capacity (8.1 wt.% LiBH_4_), limited H_2_ release (64% of theoretical capacity), and moderate reversibility (50% from first to second cycle).

### 5.5. Mixture

Zhou et al. presented an unusual two-step infiltration method: by infiltrating firstly LiBH_4_ at 300 °C and then LiAlH_4_, they managed to prevent the decomposition of the later. From the second cycle on, they obtained a somewhat fully reversible material, which they attributed to the formation of AlB_2_ [139]. After the first cycle, the decomposition curve is changed from two-steps to one and requires high temperatures of desorption (450 °C). It is striking that in comparison, the absorption occurs under moderate conditions (350 °C, 60 bar H_2_).

Utke et al. proposed several materials doped with coordination metals [140,141]. They contributed highly to the field of hydride mixture, with the nanoconfinement of LiBH_4_-MgH_2_ [114,118,119]. Interestingly, they initially obtained this mixture by a two-step MgBu_2_ wet-infiltration and reduction followed by LiBH_4_ melt infiltration [118] and later proposed a one-step milled-MgH_2_-LiBH_4_ melt infiltration [119]. The later required materials of relatively large pore size, probably due to the size of MgH_2_ nanoparticles that are not melting with LiBH_4_, thus just being dragged by it instead. The high density of MgH_2_ allow their material to display excellent wt.% H_2_ release, even at moderate temperature (320 °C), but very slowly (10 h). The main interest of this material is its excellent reversibility, although it needs high temperature (425 °C) to obtain reasonable desorption time (2–20 h) and very high pressure of rehydrogenation (140 bar H_2_).

It should be noted that the dehydrogenation was performed under relatively high hydrogen pressure (3.5 bar H_2_). The authors explained this was supposed to avoid the formation of the Li_2_B_12_H_12_ intermediate while favoring the formation of MgB_2_ [142]. A comprehensive review of the mechanism of their materials was also proposed [143]. There, they compared the two infiltration methods (one-step melt and two steps wet-melt) and obtained very similar behaviors; slightly better results were obtained when MgH_2_ was introduced by wet infiltration of Mg-Bu_2_, however. In the following work, they improved the kinetic behavior of this nanoconfined hydride mixture by the addition of TiCl_3_ [142].

Javadian et al. also proposed to impregnate LiBH_4_ with CaBH_4_. They employed a eutectic of these materials, which presents a melting point at 200 °C [144]. The reactive hydride mixture (RHM) was infiltrated at 190 °C under 130 bar H_2_ in CAS and CO_2_-activated CAS. Interestingly, they stated that CO_2_-activation might promote the formation of a graphene-like material, but the performance improvement was not explicitly attributed to this structure. While CO_2_ activation enhances the total pore volume drastically, it also increases the decomposition temperature of the nanoconfined material. However, in the pristine matrix, the nanoconfined hydride releases much less hydrogen per mass than its bulk counterpart, while in the CO_2_ activated matrix, values of liberated H_2_ are comparable to the bulk material. Similarly, the hydride confined in the pristine matrix presents the worst reversibility, while in bulk and CO_2_ activated matrix, the hydride presents similar reversibility. From these observations, the authors concluded that the LiBH_4_/CaBH_4_ might react with CAS (while LiBH_4_ does not), whereas the CO_2_ activated matrix is more inert than the non-activated one. They also presented the properties of nanoconfined LiBH_4_/NaBH_4_ [145].

Li and Chen studied the nanoconfinement of LiBH_4_-NH_3_ in SiO_2_ [146] and Al_2_O_3_ scaffolds [147]. LiBH_4_ was melt infiltrated at very low temperatures, and the compound presented excellent first cycle characteristic temperatures, yet, as for oxidized scaffolds, the reversibility is probably absent.

Sofianos et al. proposed a novel Al scaffold by sintering pressed NaAlH_4_ [148]. Despite the large pore size and its broad distribution, very low onset temperature was observed (<100 °C), suggesting that the synergies between nanoconfinement and chemical enhancement were effective. Interestingly, TiCl_3_ was initially present to decompose NaAlH_4_ but is also very likely to act as a catalyst for the decomposition of LiBH_4_. It should also be noted that the scaffold presents a very low surface area (6 m^2^ g^−1^), and most of its pore volume is due to macropores (1.12 cm^3^ g^−1^), with only a tiny portion of mesopores (0.01 cm^3^ g^−1^). The mass amount of H_2_ released is meager (0.4 wt.%) but obtained below 260 °C. In another publication, they used a mixture of different hydrides, notably 0.725LiBH_4_-0.275KBH_4_. They also obtained Al scaffolds by electrosynthesis in ionic liquid and Mn scaffolds by sintering NaMgH_3_ [149,150].

### 5.6. Performances

Among the best material obtained, none achieved all the goals for hydrogen storage applications, but each has its one strength. Cahen et al. obtained very high gravimetric mass and low-temperature release at the cost of reversibility and (maybe) kinetic behavior [112]. Gosalawit-Utke et al. obtained materials of good gravimetric mass extremely reversible but which need high temperatures and a long time [119]. The materials proposed by Liu et al. have excellent kinetic behavior but low gravimetric capacity [109]. Despite having mostly pores of significant size, Sofianos et al. reported very low onset values for their materials. This low onset is probably due to the impregnated smaller pores, while the bigger deviate lowly from the properties of the bulk material, as supported by the importance of low filling values over dehydrogenation temperatures [149]. Table 8 and Table 9 thoroughly summarize the hydrogen storage properties of nanoconfined LiBH_4_-based composites with different types of matrices (SWNT, CMK, AC, NPC, HSAG, ACNF, PMMA-BM, SBA15) and a matrix of carbon aerogel scaffolds (CAS), respectively.

## 6. Summary and Conclusions

LiBH_4_ is still one of the most promising light complex hydrides due to its large density of hydrogen (18.5 wt.% and 121 kgH_2_/m^3^). However, only 75% of its entire hydrogen density is available due to the formation of LiH and free B upon decomposition. The reversibility of pristine LiBH_4_ demands harsh conditions (>600 °C and >100 bar H_2_) [1,41], which makes it impossible for its direct use as a hydrogen storage medium for practical applications. Several investigations have been done heading towards thermodynamic destabilization and kinetic improvement of LiBH_4_ [29,30,39,46,49,50,53,58,59,60,61,62,63,64,65,66,67,69,72,73,74,75,76,77,78,79,80,81,82,83,84,85,86,87,89,90,91,92,93,94,95,96,97,98,99,101,102,103,104,105,106,107,108,109,110,111,112,113,114,115,116,117,118,119,120,121,122,123,124,125,126,127,128,129,130,131,132,133,134,135,136,137,138,139,140,141,142,143,144,145,146,147,148,149,150,151,152,153].

**Figure 5:**
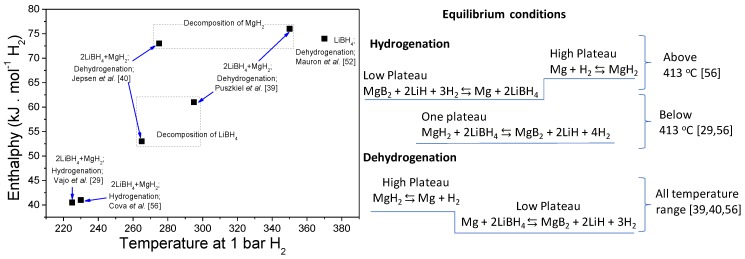
Thermodynamics of the 2LiBH_4_:MgH_2_ hydride system: reaction enthalpy as a function of the desorption temperature at 1 bar H_2_ and reaction pathways in equilibrium conditions.

One of the main approaches has been the mixture of LiBH_4_ with a broadly studied binary hydride sich as MgH_2_. Characterizations of the thermodynamic behavior for the 2LiBH_4_:MgH_2_ evidence different reaction pathways upon hydrogenation and dehydrogenation. As seen in Figure 5, the hydrogenation enthalpy shows a marked reduction of stability in comparison to pristine LiBH_4_. Moreover, for the hydrogenation under equilibrium conditions, there are two temperature regions: above and below 413 °C. On the one hand, below this temperature, just one plateau can be observed belonging to the mutual LiBH_4_ and MgH_2_ destabilization. On the other hand, above 413 °C, two plateaus are corresponding to the formation of LiBH_4_ from LiH and MgB_2_ (lower plateau) and then the hydrogenation of Mg (higher plateau). The dehydrogenation under equilibrium conditions always exposes two plateaus for all temperature range: dehydrogenation of MgH_2,_ the higher plateau, and dehydrogenation of LiBH_4_ into MgB_2_ and LiH for the lower plateau. In the case of dehydrogenation, the destabilization effect is upon LiBH_4,_ and the enthalpy reduction is not as substantial as for the hydrogenation [29,39,40,56].

**Figure 6:**
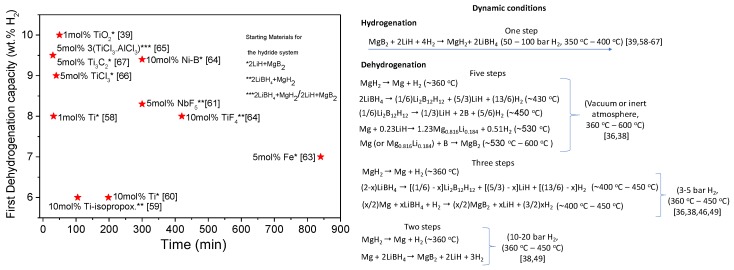
Kinetics of the 2LiBH_4_:MgH_2_ hydride system: First dehydrogenation capacities after adding TM and TMC against time for the hydrogen release and reaction pathways under dynamic conditions. ★ First dehydrogenation capacity. Starting material for the hydride system: * 2LiH + MgB_2_; ** 2LiBH_4_ + MgH_2_; *** 2LiH + MgB_2_/2LiBH_4_ + MgH_2_.

Under dynamic conditions (Figure 6), the mutual destabilization effect is only seen upon hydrogenation by following one reaction step. Upon dehydrogenation, the reaction pathways are highly dependent on the hydrogen backpressure employed during the process. Besides, several steps are depending on the temperature conditions such as those shown under a vacuum or inert atmosphere. In general, experiments to evaluate the kinetic behavior of 2LiBH_4_:MgH_2_ are performed between 3–5 bar of hydrogen backpressure and at 400 °C, conditions at which the formation of the stable species Li_2_B_12_H_12_ is barely avoided. However, the hydrogen capacity and times reached for the dehydrogenation and rehydrogenation are determined by the kind of additive used and the nature of the starting materials. As seen in Figure 6, Ti containing additives added to 2LiH + MgB_2_ show excellent performance with capacities over 8 wt %, and times are ranging between 30 min to 50 min. These results evidence an enormous reduction from the 10 to 15 h required for the first decomposition of the pristine 2LiBH_4_:MgH_2_, though the temperature condition is still quite high for an application [36,38,39,46,49,58,59,60,61,62,63,64,65,66,67].

**Figure 7:**
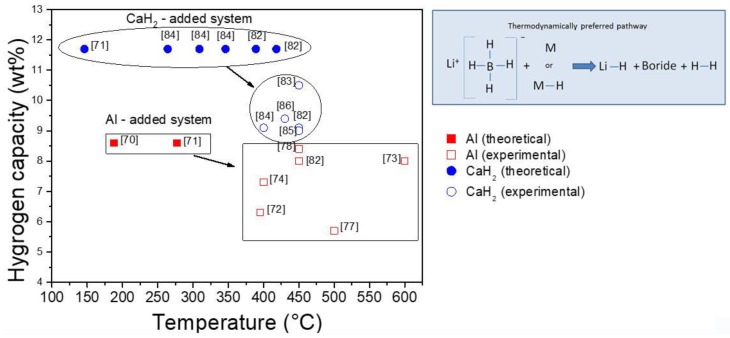
Theoretical and experimental values of hydrogen capacities (wt.%) against the temperature for hydrogen release (°C) for the LiBH_4_-Al and LiBH_4_-CaH_2_ hydride systems.

Destabilization of LiBH_4_ has also been attempted by adding metals such as Al, Mg, Ti, V, Cr, Sc, Ni, Ca, In and Fe, or other binary hydrides such as AlH_3_, TiH_2_, VH_2_, ScH_2_, CrH_2_, CaH_2_. As shown in the experimental evidence of the interactions between LiBH_4_ and metals, and binary metal hydrides have shown that Ni, Ca, and NaH form irreversible metal borides, while Cr, V, Sc, Ti, and TiH_2_ only act as a catalyst. However, Mg, MgH_2_, Al and CaH_2_ act as destabilizing agents for LiBH_4_. As seen before, the destabilization effect of Mg/MgH_2_ has been already discussed; thus, other interesting destabilization agents are Al and CaH_2_ owing to the reversibility of the hydride systems. Figure 7 shows that the thermodynamically preferred pathway for the reversible interaction between LiBH_4_ and metals, and binary hydrides upon dehydrogenation is the one leading to the formation of LiH and boride species, with hydrogen release. For the case of Al-LiBH_4_ system, the theoretical calculations predicted a hydrogen capacity of 8.6 wt.% at 188 °C and 277 °C as shown in Figure 7. Nonetheless, the measured capacities and temperatures for the Al-LiBH_4_ system range between 8.4 and 5.7 wt.% H_2_, and 395 °C and 600 °C, respectively. Theoretical calculations for the LiBH_4_-CaH_2_ system provided more promising results with a higher hydrogen capacity of 11.7 wt.% and temperatures from 146 °C to 418 °C, Figure 3. Experimental results for this system present lower capacities between 9 wt.% and 10.5 wt.% H_2_ and higher temperatures in the range of 400 °C and 450 °C. In both cases, the theoretical values are not too far away from the experimental ones, but other experimental features such as sluggish kinetic behavior and limited reversibility are still critical issues to be improved [70,71,72,73,74,78,82,83,84,85,86].

**Figure 8:**
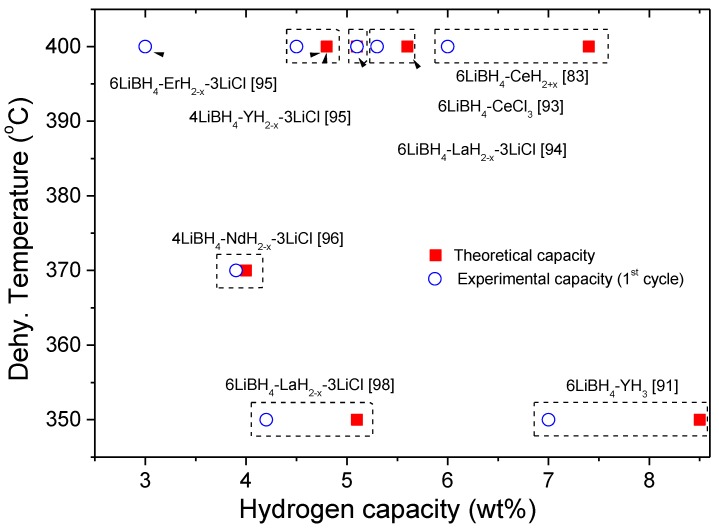
Experimental dehydrogenation temperatures vs. theoretical and experimental capacities of the LiBH_4_-RE hydride system.

Rare earth (RE) metal hydrides have also been used to destabilize LiBH_4_. Most of the used RE hydrides are not commercial, they are synthesized in house. Three approaches for the synthesis of RE metal hydride have been used: (1) RE + H_2_ in a closed-reactor at a defined temperature and pressure, (2) the mechanochemical process of RECl_3_ + LiH, and (3) decomposition of RE borohydrides. The addition of RE hydrides to LiBH_4_ is carried out by the milling process. The second synthesis approach is usually applied, hence obtaining a hydride system composed of as-milled LiBH_4_ + RE hydride + LiCl. Upon dehydrogenation, the decomposition undergoes a multi-step process until the formation of RE borides and LiH. Therefore, the general preferred thermodynamic pathway follows the one shown in Figure 7. Nanostructure, hydrogen backpressure, mainly for LiBH_4_-CeH_2_ and LiBH_4_-YH_3_, and the addition of catalysts improve the kinetic performance of the LiBH_4_- RE hydride systems. In Figure 8, the dehydrogenation temperature and the theoretical and experimental capacities of the LiBH_4_–RE hydride systems are shown. As seen, the experimental dehydrogenation temperatures range between 350 °C and 400 °C, which represent milder temperature conditions than as-milled LiBH_4_. Furthermore, in several cases, the theoretical hydrogen capacity is similar or is nearby to the experimental ones, which are not higher than 7 wt.% H_2_. In this regard, the temperature is still harsh and the capacities are lower than the above mentioned destabilized system [90,92,94,95,96,97,99]. Albeit, the LiBH_4_-RE hydride systems exhibit slightly weaker characteristics; this destabilization approach offers an alternative to be combined with other destabilizing agents, for instance, changing the stoichiometric compositions of the hydride systems, or to be used for the nanoconfinement approach.

**Figure 9:**
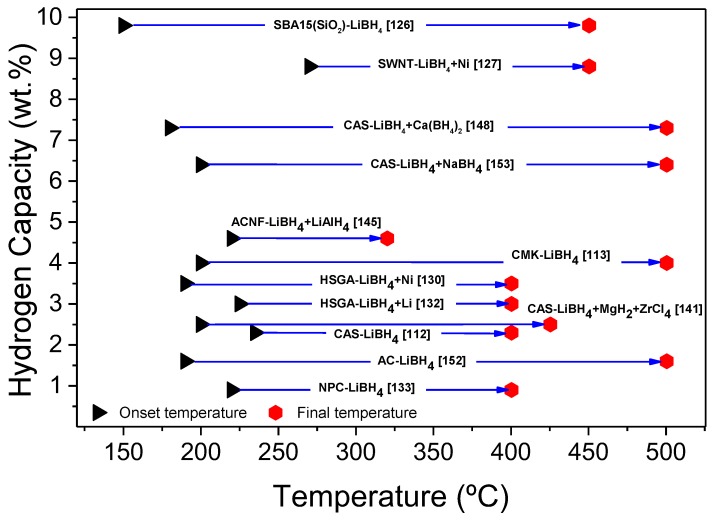
Experimental hydrogen capacity against the temperature range for the first dehydrogenation for several nanoconfined metal/metal compound added LiBH_4_ and LiBH_4_+binary/complex hydride systems.

The nanoconfinement of LiBH_4_ and LiBH_4_ + Metal/Metal hydride systems is an approach intensively explored as an option to achieve destabilization and/or kinetic improvement. The nanosized cavities of the matrix mainly prompt hydrogen exchange and notably shortens the diffusion paths. Suwarno highlighted the crucial role of the matrix other than the “activation” layer of nanoconfined hydrides. He gave a good insight into the optimized pore size for each matrix to prevent the presence of “bulk” LiBH_4_ [110]. Mason proposed a mechanism to sustain the infiltration of LiBH_4_ within a C matrix and also highlighted the role of this matrix in the ejection of LiH, reducing the reversibility of the system by promoting phase segregation [115]. These studies are highly instructive and should serve as a beacon to the experimentalist in order to design useful materials. While the use of inert matrices was fundamental to understand the mechanism of nanoconfinement, they proved LiBH_4_ would hardly attain reasonable characteristics by the sole physical constraint effect. Nanoconfinement should be enhanced by chemical means such as doping the matrix with a catalyst [133] or destabilizing the hydride with metals [144]. Still, matrices of heteroatoms are relatively rare, while they often present remarkable properties [138,150]. Finally, it is very prejudicial to the specialty that such zoology of conditions exists, which hinders the rational compilation of the results. We believe Utke et al. rightfully highlighted in their work that both desorption (3.4 bar) and absorption (100 bar) pressure plays a substantial mechanical role over LiBH_4_ decomposition and affects its reversibility [119,140]. Figure 9 shows the hydrogen capacity against the temperature range for the first dehydrogenation for several nanoconfined metal/metal compound added LiBH_4_ and LiBH_4_+binary/complex hydride systems [112,113,126,127,130,132,133,141,145,150,152,153]. As seen, the onset temperature for the dehydrogenation of the nanoconfined systems is notably reduced in comparison with the observed temperature for pristine LiBH_4_ and the LiBH_4_ destabilized systems. However, the range of temperatures for the hydrogen release process is quite large, reaching in most of the cases temperatures over 400 °C. The hydrogen capacities are mostly below 6 wt.% and just a few systems are in the range between 8 wt.% and 9 wt.% H_2_. Furthermore, a considerable loss of capacity is noticed during the second dehydrogenation in all cases (see Table 8).

Out of all achievements, much effort was made to enhance these capabilities by slowly enriching the physical and chemical nature of the materials. All in all, the most critical characteristic should be the reversibility and the economical/ecological cost of the material. In this aspect, the nanoconfinement of MgH_2_-2LiBH_4_ is appealing.

## Figures and Tables

**Table 1:** Reaction pathways for the 2LiBH_4_−MgH_2_ system: hydrogenation and dehydrogenation processes under dynamic or equilibrium conditions.

Starting Material (Catalyst/Composite Stoichiometry)	Process	Equilibrium/Dynamic Conditions Temperature and Pressure Conditions	Reaction Pathway	Ref.
Ti-isopropox. added MgB_2_:2LiH	Hydrogenation	Dynamic conditions/250–300 °C, 50 bar H_2_	MgB_2_ + 2LiH + 4H_2_ → MgH_2_ + 2LiBH_4_	[33]
MgH_2_:2LiBH_4_	Dehydrogenation	Dynamic conditions/~400 °C, 3–5 bar H_2_	MgH_2_ + 2LiBH_4_ → Mg + 2LiBH_4_ + H_2_ → MgB_2_ + 2LiH + 4H_2_	
TiCl_3_ added MgH_2_:2LiBH_4_	Dehydrogenation	Dynamic conditions/<400 °C, <3 bar H_2_	MgH_2_ + 2LiBH_4_ → Mg + 2LiBH_4_ + H_2_ → Mg + 2B + 2LiH + 4H_2_	[34]
		Dynamic conditions/280–450 °C, 3–5 bar H_2_	MgH_2_ + 2LiBH_4_ → Mg + 2LiBH_4_ + H_2_ → MgB_2_ + 2LiH + 4H_2_	
2LiH:MgB_2_ (milled 120 h)	Hydrogenation	Dynamic conditions/ramp of temp. of 2 °C/min from RT to 265 °C and then 5 h isothermal/90 bar H_2_	MgB_2_ + 2LiH + 4H_2_ → MgH_2_ + 2LiBH_4_	[35]
	Dehydrogenation	Dynamic conditions/ramp of temp. of 2 °C/min from RT to 265 °C and then 5 h isothermal/0.01 bar H_2_	MgH_2_ + 2LiBH_4_ → Mg + 2LiBH_4_ + H_2_ → MgB_2_ + 2LiH + 4H_2_	
1LiBH_4_:4MgH_2_	Dehydrogenation	Dynamic conditions/ramp of temp. of 10 °C/min from RT to 600 °C/vacuum	MgH_2_ + 0.3LiBH_4_ → 0.37Li_0.184_Mg_0.816_ + 0.15MgB_2_ + 0.78Li_0.30_Mg_0.70_ + 1.6H_2_	[36]
MgH_2_:2LiBH_4_	Dehydrogenation	Dynamic conditions/ramp of temp. of 5 °C/min from RT to >450 °C/<3 bar H_2_	MgH_2_ + 2LiBH_4_ → Mg + 2B + 2LiH + 4H_2_	[37]
		Dynamic conditions/ramp of temp. of 5 °C/min from RT to 400 °C/5 bar H_2_	MgH_2_ + 2LiBH_4_ → MgB_2_ + 2LiH + 4H_2_	
2LiBH_4_:1MgH_2_	Dehydrogenation	Dynamic conditions/ramp of temp. of 5 °C/min from RT to 600 °C/1 bar He	MgH_2_ → Mg + H_2_ 2LiBH_4_ → (1/6)Li_2_B_12_H_12_ + (5/3)LiH + (13/6)H_2_ Mg + 0.23LiH → 1.23Li_0.184_Mg_0.816_ + 0.51H_2_ Mg (or Li_0.184_Mg_0.816_) + B → MgB_2_	[38]
		Dynamic conditions/ramp of temp. of 5 °C/min from RT to 600 °C/5–10 bar H_2_	MgH_2_ → Mg + H_2_ (2 − x)LiBH_4_ → (1/6 − x)Li_2_B_12_H_12_ + (5/3 − x)LiH + (13/6 − x)H_2_ (x/2)Mg + xLiBH_4_ → (x/2)MgB_2_ + xLiH + (3/2)xH_2_	
		Dynamic conditions/ramp of temp. of 5 °C/min from RT to 600 °C/20 bar H_2_	MgH_2_ + 2LiBH_4_ → MgB_2_ + 2LiH + 4H_2_	
2LiBH_4_:1MgH_2_	Dehydrogenation	Dynamic conditions/ramp of temp. of 30 °C/min from RT to 450 °C/10 bar H_2_	MgH_2_ + 2LiBH_4_ → Mg + 2LiBH_4_ + H_2_ → MgB_2_ + 2LiH + 4H_2_	[55]
Ni added 2LiBH_4_:1MgH_2_	Hydrogenation	Equilibrium condition/375–475 °C	Above 413 °C Low plateau MgB_2_ + 2LiH + 3H_2_ → Mg + 2LiBH_4_ High Plateau Mg + H_2_ → MgH_2_ Below 413 °C MgB_2_ + 2LiH + 4H_2_ → MgH_2_ + 2LiBH_4_	[56]
	Dehydrogenation	Equilibrium condition/340–450 °C	High plateau MgH_2_ + 2LiBH_4_ → Mg + 2LiBH_4_ + H_2_ Low plateau Mg + 2LiBH_4_ → MgB_2_ + 2LiH + 3H_2_	[39]
TiO_2_ added 2LiBH_4_:1MgH_2_	Dehydrogenation	Equilibrium condition/350–425 °C		
TiCl3 added 2LiBH_4_:1MgH_2_	Dehydrogenation	Equilibrium condition/350–425 °C		[40]

**Table 2:** Experimental hydrogen storage characteristics for the 2LiBH_4_−MgH_2_/2LiH + MgB_2_ system.

Additive	First Dehydrogenation	First Hydrogenation	Cycling	Ref.
1 mol% TiF_3_ *	~8 wt.%, 400 °C, 3 bar H_2_/32 min	7.6 wt.%, 350 °C, 75 bar H_2_/4 h	Not available	[58]
10 mol% Ti-isopropox. **	~6 wt.%, 400 °C, 5 bar H_2_/1.75 h	~6 wt.%, 350 °C, 50 bar H_2_/4.5 h	Not available	[59]
10 mol% Ti *	~6 wt.%, 400 °C, 3 bar H_2_/3.3 h ^(1)^	9.5 wt.%, 400 °C, 50 bar H_2_/3.3 h	Not available	[60]
5 mol% NbF_5_ **	8.3 wt.%, 400 °C, 4 bar H_2_/5 h	8.2 wt.%, 400 °C, 65 bar H_2_/30 min	15 cycles/Hydro. average: 8.9 wt.%/Dehydro. average: 8.3 wt.%	[61]
10 wt.% Ni-B *	9.4 wt.%, 400 °C, 4 bar H_2_/5 h	Not available	3 cycles/Av. dehydro. capacity: ~9 wt.%	[62]
5 mol% Fe *	~7 wt.%, 400 °C, 5.5 bar H_2_/14 h	~7 wt.%, 350 °C, 50 bar H_2_/4 h	3 cycles/Loss of capacity of about 0.5 wt.%	[63]
1 mol% TiO_2_ *	~10 wt.%, 400 °C, 3 bar H_2_/50 min	~10 wt.%, 400 °C, 50 bar H_2_/25 min	10 cycles/stable	[39]
10 mol% TiF_4_ **	~8 wt.%, 390 °C, 4 bar H_2_/7 h	~8 wt.%, 350 °C, 50 bar H_2_/3 h	Not available	[64]
~5 mol% (3TiCl_3_. AlCl_3_) *^/^**	~9.5 wt.%, 400 °C, 4 bar H_2_/30 min	~9.5 wt.%, 350 °C, 100 bar H_2_/30 min	25 cycles/Loss of capacity ^(2)^: 0.061 wt.%/cycle/0.039 wt.%/cycle	[65]
5 mol% TiCl_3_ * ^(3)^	~9 wt.%, 400 °C, 2 bar H_2_/40 min	~9 wt.%, 350 °C, 50 bar H_2_/10 h	20 cycles/Loss of capacity: 0.002 wt.%/cycle	[66]
5 wt.% Ti_3_C_2_ *	~9.5 wt.%, 390 °C, 3 bar H_2_/30 min	~ 9.5 wt.%, 350 °C, 50 bar H_2_/5 min	15 cycles/8% of capacity reduction (after cycling: 8.7 wt.%)	[67]

* Starting material: 2LiH + MgB_2_. ** Starting material: 2LiBH_4_ + MgH_2_. ^(1)^ Dehydrogenation not finished. ^(2)^ For the starting materials in the hydrogenated estate: 0.061 wt.%/cycle and for the starting materials in the dehydrogenated estate: 0.039 wt.%/cycle. ^(3)^ 500 mg of sample/time to reach 80% of the full capacity.

**Table 3:** Calculated and experimental hydrogen storage properties of LiBH_4_-based systems.

	Additive	Theoretical and Predicted Values				Experimental Values		
		Predicted Reaction	H_2_ Content (wt.%)	ΔH^300 K^ (kJ/mol H_2_)	T, P = 1 bar (°C)	Temperature of H_2_ Release (°C)	Experimental wt.%	Conditions (T, P) for 1° Isothermal Desorption ^(1)^
		2LiBH_4_ → 2LiH + 2B + 3H_2_	13.9	62.8 ^(3)^	322 ^(3)^	450 ^(1)^	0 at 400 °C ^(1)^9 at 600 °C ^(2)^	-
		LiBH_4_ → Li + B + 2H_2_	18.5	89.7 ^(3)^	485 ^(3)^	-	-	-
Metals	Al	LiBH_4_ + 1/2Al → LiH + 1/2AlB_2_ + 3/2H_2_	8.6	57.9 ^(3)^	277 ^(3)^188 ^(4)^	350 and 430 ^(1)^	6.8 ^(1)^, 7.8 ^(2)^	395-1 bar
	Mg	-	-	-	-	430 ^(1)^	5.6 ^(1)^, 9 ^(2)^	375-1 bar
	Ti	-	-	-	-	405 ^(1)^	2.5 ^(1)^	400-1 bar
	V	-	-	-	-	430 ^(1)^	4.4 ^(1)^	400-1 bar
	Cr	2LiBH_4_ + Cr → CrB_2_ + 2LiH + 3H_2_	6.3	31.7 ^(3)^	25 ^(3)^	415 ^(1)^	4.4 ^(1)^	400-1 bar
	Sc	-	6.7	-	-	420 ^(1)^	2.9 ^(1)^	400-1 bar
	Ni	-	-	-	-	-	-	-
	Ca	-	-	-	-	-	5.9 ^(2)^	-
	In	-	-	-	-	-	7.8 ^(2)^	-
	Fe	2LiBH_4_ + 2Fe → 2FeB + 2LiH + 3H_2_	3.9	12.8 ^(3)^	−163 ^(3)^	-	-	-
	Fe	2LiBH_4_ + 4Fe → 2Fe_2_B + 2LiH + 3H_2_	2.3	1.2 ^(3)^	-	-	-	-
Hydrides	AlH_3_	4LiBH_4_ + 2AlH_3_ → 2AlB_2_ + 4LiH + 9H_2_	12.4	39.6 ^(3)^	83 ^(3)^	-	-	-
	TiH_2_	2LiBH_4_ + TiH_2_ → TiB_2_ + 2LiH + 4H_2_	8.6	4.5 ^(3)^	-	410 ^(1)^	1.7 ^(1)^	390-1 bar
	VH_2_	2LiBH_4_ + VH_2_ → VB_2_ + 2LiH + 4H_2_	8.4	7.2 ^(3)^	−238 ^(3)^	-	-	-
	ScH_2_	2LiBH_4_ + ScH_2_ → ScB_2_ + 2LiH + 4H_2_	8.9	32.6 ^(3)^	26 ^(3)^	-	-	-
	CrH_2_	2LiBH_4_ + CrH_2_ → CrB_2_ + 2LiH + 4H_2_	8.3	16.4 ^(3)^	−135 ^(3)^	-	8.3 ^(2)^	-
	CaH_2_	6LiBH_4_ + CaH_2_ → CaB_6_ + 6LiH + 10H_2_	11.7	45.4 ^(3)^	146 ^(3)^	415 ^(1)^	5.1 ^(1)^, 9 ^(2)^	395-1 bar
	MgH_2_	2LiBH_4_ + MgH_2_ → MgB_2_ + 2LiH + 4H_2_	11.4	50.4 ^(3)^	186 ^(3)^170 ^(4)^	350 and 430 ^(1)^	10.2 ^(1)^, 7.8 ^(2)^	370-1 bar

^(1)^ 1° isothermal desorption at 400 °C [72]/^(2)^ TPD up to 600 °C [73]/^(3)^ [71]/^(4)^ [70].

**Table 4:** Experimental H_2_ properties for the LiBH_4_-Al system.

Ref.	Composition	H_2_ wt.% at Successive Cycles of Dehydrogenation	Type of Measurement	Temperature, Pressure, and Operating Time Conditions for Rehydrogenation
[72]	LiBH_4_ + 0.2Al	6.3, 4.2, 3.8, 5.1, 6.7	Isothermal dehydrogenation at 395 °C, 1 bar Isothermal dehydrogenation at 395 °C, 3 bar	350 °C, 150 bar, not available
[73]	LiBH_4_ + 0.5Al	8, 3.5	TPD up to 600 °C, vacuum	600 °C, 100 bar, not available
[74]	LiBH_4_ + 0.5Al + 0.04 TiF_3_	7.3, 5.1, 4.1, 3	Isothermal dehydrogenation at 400 °C, 1 bar	400 °C, 100 bar, 100 min
[75]	LiBH_4_ + 0.5Al	8, 7, 2.5	PCI 450 °C	500 °C, 150 bar, 1200 min
[77]	LiBH_4_ + 1.5Al LiBH_4_ + 1.5Al + 0.045 TiB_2_	5.7, 4.2, 3.6, 3, 2.7, 2.5, 2.4, 2.2, 2, 1.8 5.5, 4.4, 3.4, 2.9, 2.5, 2, 2.2, 1.9, 1.8, 1.6	No isothermal desorption up to 500 °C, 0.01 bar	400 °C, 100 bar, 120 min
[78]	LiBH_4_ + 0.5Al + 0.04 TiCl_3_	8.4, 5.8	TGA up to 450 °C	380 °C, 150 bar, 1080 min

**Table 5:** Calculated and experimental thermodynamic information. ^1^ Scientific Group Thermodata Europe, ^2^ Ultra-Soft Pseudo-Potentials, ^3^ Projector-Augmented Wave.

Ref.		Method	ΔH (kJ mol^−1^)	T _P = 1bar_ (°C)
[82]	Calculation	HSC Chemistry HSC Chemistry with modified parameters for LiBH_4_	66.2 59.2	389 418
[84]		Thermo-Calc (SGTE ^1^ database) Thermo-Calc (SGTE database with modified parameters for CaB_6_)	60.9 48.8	346 264
[88]		Ab initio Simulation USPP ^2^—Hexagonal LiBH_4_ Ab initio Simulation USPP—Orthorhombic LiBH_4_	50.4 60.3	
		Ab initio Simulation PAW ^3^—Hexagonal LiBH_4_ Ab initio Simulation PAW—Orthorhombic LiBH_4_	52.9 62.7	
[84]	Experimental	Measured equilibrium pressures	56.5	309

**Table 6:** Different synthesis routes of rare earth (RE) hydrides.

Synthesis Procedure	Destabilized LiBH_4_ Based Composite	Ref.
Gas-solid reaction (pressure and temperature) RE_(s)_ + (2 + x)/2 H_2(g)_ → REH_2+x(s)_	RE^3+^ = Sc, Ce, Y	[54,83,89,90,91,92]
Solid-solid reaction (ball milling) RECl_3(s)_ + 3LiH_(s)_ → REH_2+x(s)_ + 3LiCl + (1 − x)/2 H_2(g)_	RE^3+^ = Ce, La, Nd	[94,95,96]
Ball milling followed by heating LiRE(BH_4_)3Cl → (1 − 3/m)REH_n(s)_ + LiCl + 3/mREB_m(s)_+ [(6 − n/2)+(3n/2m)]H_2(g)_ RE(BH_4_)_3(s)_ → (1 − 3/m)REH_n(s)_ + 3/mREB_m(s)_ + [(6 − n/2) + (3n/2m)]H_2(g)_	RE^3+^ = Ce, Gd, La, Pr, Nd, Sm	[93,94,97,98,99,103,104,105]
	RE3+ = Y, Sm, Eu, Gd, Tb, Er, Yb and Lu	[95,97,98,99,106,107]

**Table 7:** Hydrogen storage properties of LiBH_4_-based composites destabilized by interaction with RE hydrides produced using different procedures.

Composite	Solid Products	Theoretical Capacity (wt.%) *	Experimental Capacity (wt.%) 1st Cycle/2th Cycle	Experimental Conditions Rehyd./Dehyd.	Ref.
6LiBH_4_-CeH_2+x_	CeB_6_ + 6LiH	7.4	6.0/6.0	350 °C, 100 bar/400 °C, vacuum	[83]
4LiBH_4_-YH_3_	YB_4_ + 4LiH	8.5	7.0/5.2	350 °C, 90 bar/350 °C, 5 bar	[91]
6LiBH_4_-CeCl3	CeB_6_ + 3LiH + 3LiCl	5.6	5.3/2.3	400 °C, 60 bar/400 °C, 0.2 bar	[93]
6LiBH_4_-GdCl_3_	GdB_4_ + 3LiH + 3LiCl + B	5.3	5.0/2.0	400 °C, 60 bar/400 °C, 0.2 bar	
6LiBH_4_-CeH_2+x_-3LiCl	CeB_6_ + 6LiH + 3LiCl	5.1	4.6/4.6	400 °C, 60 bar/400 °C, 0.2 bar	[94]
6LiBH_4_-LaH_2+x_-3LiCl	LaB_6_ + 6LiH + 3LiCl	5.1	5.1/3.6	400 °C, 60 bar/400 °C, 0.2 bar	
4LiBH_4_-YH_2+x_-3LiCl	YB_4_ + 4LiH + 3LiCl	4.8	4.5/4.1	400 °C, 65 bar/400 °C, 0.2 bar	[95]
4LiBH_4_-NdH_2+x_-3LiCl	NdB_4_ + 4LiH + 3LiCl	4.0	3.9/3.9	400 °C, 100 bar/370 °C, vacuum	[96]
6LiBH_4_-LaH_2+x_-3LiCl	LaB_6_ + 6LiH + 3LiCl	5.1	4.2/0.8	340 °C, 100 bar/350 °C, 5 bar	[98]
6LiBH_4_-ErH_2+x_-3LiCl	ErB_4_ + 4LiH + 3LiCl + B	4.8	3.0/2.4	340 °C, 100 bar/400 °C, 5 bar	[95]

* Calculated considering the starting mixture and the LiH formed in the products.

**Table 8:** Hydrogen storage properties of nanoconfined LiBH_4_-based composites in different matrixes.

Matrix				Filling ^f^			Destabilizing Agent		Temperatures ^d^			H_2_ Release wt.% ^b^			Pressure ^e^		Notes	Ref.
Elem	Type	Dop	P.S.	Impr	wt.% ^a^	vol%	Hydride	Additive	Onset	50% ^c^	Final	1st	2nd	3rd	D Bar	R Bar		
C	SWNT			Mec	77 *			Ni	270		450	11.4 (8.8 *)	6.1 (4.7 *)	4.6 (3.5 *)	<1 × 10^−3^	100	Catalyst from synthesis	[126]
C	CMK			Mec	50 *				225 **	332	600	14 (7)	6 (3)	ND	1	30	TPD	[128]
C	AC			Wet	30	70 *			220	300	350	11.2 (3.4) *	6.6 (2.0) *		1 × 10^−3^	50	TPD	[127]
C	AC		12 **	Melt	12 *	35			190	320 **	500	13.6 (1.6) *	6.0 (0.7) *	6.0 (0.7) *	NS	60	TPD	[151]
C	CMK		4	Wet	33	67 *			200	235 **	500	12.0 (4.0)	« 0 »	NS	<0.1	100	18 h. Impregnation in MTBE	[112]
					50	100 *			200	280 **		12.0 * (6.0)					Outer LiBH_4_	
C	NPC		2.0		10 *	50 *			220	310 **	400 **	9.0 ** (0.9) **	NS	NS				[132]
C	NPC		4.0		20	70			220 **	350	350	NS	6.9 ** (1.4) **	5.5 ** (1.1) **	1 × 10^−5^	60	Isotherm	[109]
C	HSAG		2–3		25	75 *		Ni	<200	<350	400	14 (3.5)	9.2 (2.3)	NS	1	40	TPD Ar flow	[129]
C	HSAG		2–3	Melt	20	80 *		Li	225 **	340 **	400	15 * (3.0)	10.9 * (2.2)	NS	1	60	10 wt.% LiH	[131]
C	ACNF		2.8	Wet	50	X	LiAlH_4_		220	302	320	9.2 * (4.6)	7.6 * (3.8)	6.0 * (3.0)	<1 × 10^−5^	80	No density	[152]
C	ACNF			Melt				TiO_2_									Compacted	[141]
C	PMMA-BM			Wet	8.1	X			80	105	120	0.74 (8.8)	0.31 (3.8)	NS	vacuum	50	No pore size information	[138]
Al			1.7–50+	Melt	10.5	5 *		TiCl_3_	380	440 **	540	(1.8)	NS	NS	0.88		TPD LiAl formed	[148]
					14.5	8 *			100	490 **	540	(2.8)	NS	NS	1.3		TPD	
					21.4	12 *			180 **	240 **	265	2.0 ** (0.42) **	1.6 ** (0.34) **	1.2 ** (0.26) **	0.82	80		
					27.4	17 *			350	480 **	540	(3.8)	NS	NS	1.4			
Al				Melt	30	X	KBH_4_	TiCl_3_	100	450 **	510	7.3 * (2.2)					TPD No pore size information	[153]
Al_2_O_3_			6.2	Melt	20	15 *	NH_3_		65	140 **	280	14.4 ** (2.9) **	NS	NS	1	NS	TPD	[147]
					33	30 *			65	160 **	280	12.9 ** (4.2) **	NS	NS	1	NS		
					50	60 *			65	190 **	280	8.8 ** (4.4) **	NS	NS	1	NS		
Mg			1.7–50+	Melt	12.8				100	465	550	21.8 * (2.8)	NS	NS	<1.2		TPD LiH+Mg →LiMg + 0.5H_2_	[148]
					22.5				100	490	550	22.7 * (5.1)	NS	NS	<2.5			
					32.6				100	490	550	21.8 * (7.1)	NS	NS	<2.9			
SiO_2_	SBA15		5–9	Melt	40 *	100			150	295 **	450	15.0 ** (9.8) **	3.7 ** (2.4) **	2.8 ** (1.8) **	0.13	100		[125]
SiO_2_			11 **	Melt	33	66 *	NH_3_		80 **	130 **	300	8.8 * (5.8)*	NS	NS	1	NS	TPD	[146]

^a^ LiBH_4_/material; ^b^ H_2_/LiBH_4_ (H_2_/material); ^c^ or first intense TPD peak; ^d^ first hydrogen release; ^e^ pressures of dehydrogenation (D) and rehydrogenation (R); ^f^ filling method (impregnation) and values (in weight and in volume). * calculated (the value is not explicitly given by the authors but is obtained from values explicitly stated); ** estimated (the value is obtained from graphical interpretation, or calculated from at least one value obtained by graphical interpretation). SWNT: single-walled nanotube, ACNF: activated carbon nanofibers, HSAG: high surface area graphite, NPC: nanoporous carbon, AC: activated carbon or charcoal; NS: not stated; Ff: furfural; SBA: santa barbara amorphous material.

**Table 9:** Hydrogen storage properties of nanoconfined LiBH_4_-based composites in CAS matrix.

Matrix				Filling ^f^			Destabilizing Agent		Temperatures ^d^			H_2_ Release wt.% ^b^			Pressure ^e^		Notes	Ref.
Elem	Type	Dop	P.S.	Impr	wt.% ^a^	vol%	Hydride	Additive	Onset	50% ^c^	Final	1st	2nd	3rd	D Bar	R Bar		
C	CAS		13	Melt	27 *	70 *			230		300	12.6 (3.5)	8.2 * (2.3) *	6.9 * (1.9) *	<0.05	100	Impregnation under Ar	[113]
C	CAS	G	16	Melt	17 *	30			235 **	325 **	400	13.8 (2.3) *	6.5 ** (1.1) **	NS	0.5	60		[111]
					32 *	70			245 **	340 **	400	13.8 (4.4) *	6.8 ** (2.2) **	NS	0.5	60		
			6.1	Melt	12 *	30			200 **	325 **	400	13.8 (1.7) *	6.2 ** (0.7) **	NS	0.5	60		
					24 *	70			210 **	335 **	400	13.8 (3.3) *	7.7 ** (1.9) **	NS	0.5	60		
C	CAS	N-G	7.6	Melt	9 *	30			190 **	315 **	400	13.8 (1.2) *	6.6 (0.6) *	NS	0.5	60		[133]
					18 *	70			200 **	330 **	400	13.8 (2.5) *	6.3 (1.2) *	NS	0.5	60		
			4.2	Melt	6 *	30			180 **	310 **	400	13.8 (0.8) *	4.5 (0.3) *	NS	0.5	60		
					13 *	70			205 **	320 **	400	13.8 (1.8) *	5.3 (0.7) *	NS	0.5	60		
C	CAS	N-G	9.0	Melt	11 *	30		Ni	175 **	330 **	400	12.3 (1.3) *	7.5 (0.8) *	NS	0.5	60		[137]
					22 *	70		Ni	150 **	325 **	400	13.8 (3.0) *	7.9 (1.7) *	NS	0.5	60		
					10 *	30		Co	150 **	305 **	400	12.3 (1.2) *	4.0 (0.4) *	NS	0.5	60		
					21 *	70		Co	200 **	340 **	400	13.8 (2.9) *	6.4 (1.3) *	NS	0.5	60		
					10 *	30		NiCo	200 **	325 **	400	12.3 (1.3) *	6.3 (0.6) *	NS	0.5	60		
					21 *	70		NiCo	150 **	330 **	400	13.8 (2.9) *	6.2 (1.3) *	NS	0.5	60		
C	CAS		21	W + M	34 *	48 *	MgH_2_		260	320 **	390	11.4 * (3.9)	8.2 * (2.8)	10.6 * (3.6)	2	70; 98	20 h	[118]
C	CAS		31	Melt	33	43 *	MgH_2_		260 **	320 **	425	10.8 (3.6)	10.8 (3.6)	10.8 (3.6)	3.4	145		[119]
C	CAS	Ff	5.5	Melt	43	425 *	MgH_2_		260 **	410 **	425	7.9 * (3.4)	11.2 (4.8)	10.0 (4.3)	3.5	140	V_tot_ = 0.21 cm^3^/g 6–25 h	[114]
C	CAS		26	Melt	33	44 *	MgH_2_	TiCl_3_	260 **	370 **	425	10.8 (3.6)	9.8 (3.25)	10.8 (3.6)	3.4	140	2–8 h	[143]
C	CAS		26	Melt	25	30 *	MgH_2_	ZrCl_4_	200	295 **	425 **	(2.5)	NS	NS	3.4	130	TPD	[140]
					33	45 *			200	320 **	425	11.1 * (3.7)	10.5 * (3.5)	10.2 * (3.4)				
					50	90 *			200	340 **	425 **	(5.4)	NS	NS			TPD	
C	CAS		30	Melt	64.9	60	Ca(BH_4_)_2_		180 **	340 **	500	11.3 (7.3) *	9.1 (5.9) *	8.2 (5.3) *	1	150	CO_2_ activated	[144]
			30		38.4	60			150 **	230 **	500	6.2 (2.4) *	3.6 (1.4) *	3.2 (1.2) *	1	150		
C	CAS		38	Melt	55.5	60	NaBH_4_		200 **	340 **	500	11.5 (6.4)	7.9 (4.4) *	7.8 (4.3) *	1	150	CO_2_ activated	[145]
			37		32.8	60			210 **	410 **	500	10.5 (3.4)	6.3 (2.1) *	5.8 (1.9) *	1	150		
C	CAS		10 **	Melt	NS	NS	LiAlH_4_		100	290 **	500	11.0 * (X)	5.7(X)	5.7(X)	NS	60	TPD 2-step impregnation	[139]

^a^ LiBH_4_/material; ^b^ H_2_/LiBH_4_ (H_2_/material); ^c^ or first intense TPD peak; ^d^ first hydrogen release; ^e^ pressures of dehydrogenation (D) and rehydrogenation (R); ^f^ filling method (impregnation) and values (in weight and in volume). * calculated (the value is not explicitly given by the authors but is obtained from values explicitly stated); ** estimated (the value is obtained from graphical interpretation, or calculated from at least one value obtained by graphical interpretation). CAS: carbon aerogel scaffold.
